# Neural effects of dopaminergic compounds revealed by multi-site electrophysiology and interpretable machine-learning

**DOI:** 10.3389/fphar.2024.1412725

**Published:** 2024-07-09

**Authors:** Sampath K. T. Kapanaiah, Holger Rosenbrock, Bastian Hengerer, Dennis Kätzel

**Affiliations:** ^1^ Institute of Applied Physiology, Ulm University, Ulm, Germany; ^2^ Boehringer Ingelheim Pharma GmbH & Co. KG, Div. Research Germany, Ingelheim, Germany

**Keywords:** antipsychotics, neural connectivity, granger causality, power spectral density, electrophysiology, pharmaco-EEG, prediction-powered inference, interpretable machine learning

## Abstract

**Background:**

Neuropsychopharmacological compounds may exert complex brain-wide effects due to an anatomically and genetically broad expression of their molecular targets and indirect effects *via* interconnected brain circuits. Electrophysiological measurements in multiple brain regions using electroencephalography (EEG) or local field potential (LFP) depth-electrodes may record fingerprints of such pharmacologically-induced changes in local activity and interregional connectivity (pEEG/pLFP). However, in order to reveal such patterns comprehensively and potentially derive mechanisms of therapeutic pharmacological effects, both activity and connectivity have to be estimated for many brain regions. This entails the problem that hundreds of electrophysiological parameters are derived from a typically small number of subjects, making frequentist statistics ill-suited for their analysis.

**Methods:**

We here present an optimized interpretable machine-learning (ML) approach which relies on predictive power in individual recording sequences to extract and quantify the robustness of compound-induced neural changes from multi-site recordings using Shapley additive explanations (SHAP) values. To evaluate this approach, we recorded LFPs in mediodorsal thalamus (MD), prefrontal cortex (PFC), dorsal hippocampus (CA1 and CA3), and ventral hippocampus (vHC) of mice after application of amphetamine or of the dopaminergic antagonists clozapine, raclopride, or SCH23390, for which effects on directed neural communication between those brain structures were so far unknown.

**Results:**

Our approach identified complex patterns of neurophysiological changes induced by each of these compounds, which were reproducible across time intervals, doses (where tested), and ML algorithms. We found, for example, that the action of clozapine in the analysed cortico-thalamo-hippocampal network entails a larger share of D1—as opposed to D2-receptor induced effects, and that the D2-antagonist raclopride reconfigures connectivity in the delta-frequency band. Furthermore, the effects of amphetamine and clozapine were surprisingly similar in terms of decreasing thalamic input to PFC and vHC, and vHC activity, whereas an increase of dorsal-hippocampal communication and of thalamic activity distinguished amphetamine from all tested anti-dopaminergic drugs.

**Conclusion:**

Our study suggests that communication from the dorsal hippocampus scales proportionally with dopamine receptor activation and demonstrates, more generally, the high complexity of neuropharmacological effects on the circuit level. We envision that the presented approach can aid in the standardization and improved data extraction in pEEG/pLFP-studies.

## 1 Introduction

Symptoms of mental disorders are thought to often result from maladaptive activity in particular brain regions or from pathological communication between multiple brain areas (Millan et al., 2012; Deisseroth, 2014). Whereas the effects of neuropsychopharmacological compounds are usually well understood at the molecular level, their impact on circuit mechanisms that mediate psychological functions remains largely elusive. Electroencephalography (EEG) or local field potential (LFP) recordings during compound administration—termed pharmaco-EEG (pEEG) or pharmaco-LFP (pLFP)—may reveal neurophysiological effects at the circuit level whose patterns are specific to a given compound or are specifically shared by a representative psychopharmacological class of compounds (Itil, 1983; Dimpfel et al., 1986; Dimpfel et al., 1992; Dimpfel, 2009; Drinkenburg et al., 2016; Grotell et al., 2021). Despite their great contribution to describe certain patterns of neurophysiological effects of a large number of compounds in both humans and rodents, their translation into a mechanistic understanding regarding therapeutic action remains a challenge (Gener et al., 2019; Delgado-Sallent et al., 2022). In contrast to investigations of cognitive functions, a majority of pEEG studies still limit analysis to activity rather than including connectivity [with many notable exceptions, e.g., (Ahnaou et al., 2014; Rangel-Barajas et al., 2017; Gener et al., 2019)], despite the importance of neural communication for psychological functions and their psychiatric aberrations (Drinkenburg et al., 2016; Hultman et al., 2018; Strahnen et al., 2021b; Delgado-Sallent et al., 2022). Furthermore, it was shown in a pioneering application of unsupervised machine-learning that the inclusion of connectivity-data aids in discriminating different neuropharmacological compounds (Ahnaou et al., 2014). Additionally, just like electrophysiological investigations of psychological functions, mechanistic conclusions in pEEG are hampered by the streetlight effect (Cunniff et al., 2020), i.e., by the problem that a large proportion of drug-induced neural effects go unnoticed because they are simply not recorded or not revealed by the applied analysis. To reduce the streetlight effect in pEEG/LFP studies, more recording sites and depth electrodes (Dimpfel, 2007) (to improve spatial and temporal resolution compared to surface electrodes) may be used and multiple activity and connectivity metrics be analysed (Hultman et al., 2018; Strahnen et al., 2021a; Strahnen et al., 2021b; Delgado-Sallent et al., 2022) with a fine-grained separation of potentially independent frequency bands (Dimpfel, 2009). However, this approach vastly expands the number of neurophysiological variables to be analyzed in each pharmacological condition [combinatorial explosion (Ahnaou et al., 2014; Strahnen et al., 2021b; Delgado-Sallent et al., 2022)], which may be further amplified by the inclusion of factors such as temporal segmentation of post-injection analysis time and number of doses (Dimpfel, 2007; Dimpfel, 2009), whereas subject numbers remain typically low (on the order of 10). Such datasets are not suited for analysis with frequentist statistics, i.e., the inference based on *p*-values derived from *t*-tests, ANOVAs or similar pairwise comparisons. This is because *p*-values would need to be adjusted for multiple comparisons by division by the large number of analysed parameters, whereas statistical power remains relatively weak due to the typically small number of animals that can be tested in logistically demanding electrophysiological experiments - the combination of these two factors decreases the detectability of actual effects. Also, such statistical analysis neglects the possibility that several of the extracted parameters—e.g., power in adjacent frequency bands or connectivity involving the same brain region—may not be independent from each other (Bzdok et al., 2018).

To tackle this problem, we here suggest an interpretable machine-learning (ML) based approach to extract the most robust drug-induced changes in neurophysiological activity and connectivity obtained from multiple brain sites. In this approach, binary classifiers are trained to discriminate data recorded in distinct pharmacological conditions, e.g., drug vs. vehicle, and the predictive power of a given activity or connectivity parameter, estimated on individual short sequences of electrophysiological recordings, is used as a measure for its compound-induced modulation. Its principal logic of prediction-powered inference implies that drug-induced changes that are pronounced and robust will allow an ML algorithm to predict, if a short sequence of data was recorded from an animal receiving a given compound or its vehicle. This approach also effectively harnesses the vast amount of time-series data provided by every animal and session to quantify drug-induced changes, instead of relying on statistics on animal-based averages.

We demonstrate the feasibility of this method by extracting activity and connectivity alterations induced in a cortico-thalamo-hippocampal network by three pharmacologically distinct dopaminergic antagonists—clozapine (at two doses), raclopride, and SCH23390—and the indirect dopaminergic agonist amphetamine. Although clinically and pharmacologically important, the changes of directed communication between brain areas caused by such compounds remained unknown to date. Our data and analysis reveal and quantify complex, but reproducible changes across 20 directed neural connections and six frequency bands which represent finger-prints of every analysed compound and point to their mechanistic circuit effects.

## 2 Materials and methods

### 2.1 Animals and surgery

**FIGURE 1:**
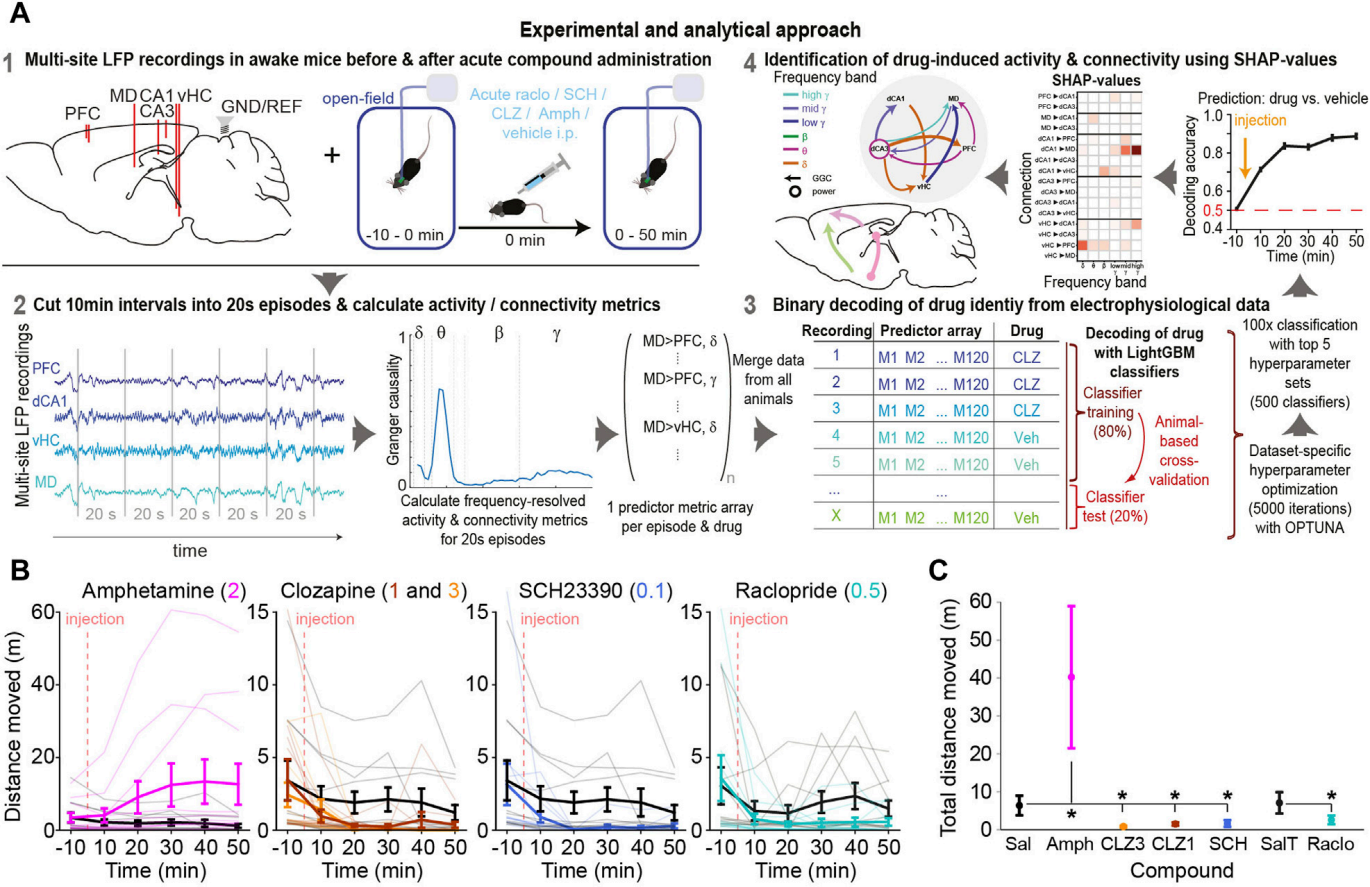
Experimental approach and drug-induced changes of behaviour and neurophysiology **(A)** LFPs were recorded from prefrontal cortex (PFC), dorsal/ventral hippocampus (dCA1, dCA3, vHC) and mediodorsal thalamus (MD) of awake mice receiving a certain compound or vehicle. Directed connectivity between these sites was calculated in 20s episodes and a Light Gradient Boosting Machine (LGBM) learning model was trained to predict which drug a mouse has received based on connectivity patterns of such episodes. SHAP-values were used to quantify the predictive power of each frequency and connection, which measures the magnitude and robustness of its compound-induced change **(B)** Locomotor activity across time in 10 min intervals around the time of injection of compound (colour, dose in mg/kg indicated at the top) or vehicle (grey); thin lines represent individual mice; thick lines and error bars represent mean ± s. e.m. **(C)** Same data as in **(B)** but total distance moved after injection summed up; asterisks indicate significant difference to corresponding vehicle (*p* < 0.05, one-sided *t*-test).

All experiments were performed in accordance with the German Animal Rights Law (Tierschutzgesetz) 2013 and were approved by the Federal Ethical Review Committee (Regierungsprädsidium Tübingen) of Baden-Württemberg, Germany (licence number TV1399). In total, two male and eight female adult (age: 4–7 months) C57BL/6N wildtype mice, which were littermates obtained from cross-breeding of heterozygous *Gria1*-knockout mice [*Gria1* ^tm1Rsp^; MGI:2178057 (Zamanillo et al., 1999)], were used for this study. Sample sizes were chosen to be around 10 based on our prior analysis of connectivity data (Strahnen et al., 2021a). Mice were operated as described previously (Strahnen et al., 2021a) and in Supplementary Methods, implanting chronic PTFE-insulated Tungsten electrodes of 50 μm diameter into five sites (Figure 1A): prelimibic cortex (PrL), mediodorsal thalamus (MD), the fissure of the dorsal hippocampus (dCA1), the CA3-subfield of the dorsal hippocampus (dCA3), the fissure of the ventral hippocampus (vHC). A scull screw above the contralateral cerebellum served as ground and reference electrode. Accurate electrode placements in the target region were verified *post-mortem* by electrolytic lesions made immediately after death, and misplaced electrodes were excluded from analysis.

### 2.2 Pharmacology and recordings

Pharmaco-LFP experiments were conducted approximately every third day in a within-subject design with a latin-square, randomized drug-assignment across the cohort. Applications included: saline (Sal; vehicle 1), 0.1% TWEEN80/saline (T/Sal; vehicle 2), 1 and 3 mg/kg clozapine (CLZ1 and CLZ3; clozapine dihydrochloride, HB6129, HelloBio, GB), 0.5 mg/kg raclopride (Raclo; S (−)raclopride (+)-tartrate, R121 Sigma, DE), 0.1 mg/kg SCH23390 (SCH; SCH23390 hydrochloride, 0925 Tocris, GB), and 2 mg/kg amphetamine (Amph; d-amphetamine sulfate, A-5880, Sigma); vehicle 2 was used for raclopride, all other compounds were applied in saline. Mice were recorded in Type-III open-field cages (Tecniplast, IT) with fresh saw-dust for a 10 min baseline period, after which the compound was applied i. p., followed by a further recording for 50 min (see Supplementary Methods; Figure 1A). Novelty-induced locomotion was used deliberately to induce a brain state with high dopamine release (and resulting exploratory motivation) to improve the detectability of behavioural and electrophysiological effects of dopamine-enhancing and -antagonizing compounds. Data was recorded with a 32-channel RHD2132 headstage for amplification and digitization (Intan Technologies, CA, United States) and an Open-Ephys (Siegle et al., 2017) acquisition system (20 kHz sampling rate). A custom-made motorized Open-MAC commutator (Kapanaiah and Kätzel, 2023) served to neutralize the torque created by the animal’s rotations.

### 2.3 Data analysis

Key design considerations for the recording and analysis approach are stated in Table 1.

**TABLE 1:** Design considerations for the analysis approach.

Analysis parameter	Problems, constraints, trade-offs	Design solution
N and subject-design	Effort for implantation and experimentation, loss of electrodes	8–10, within-subject
Mouse exclusion	Loss of data vs. low accuracy due to outlier reaction to drugs	Exclusion of extreme outliers (non-moving or hyperactive)
Electrode exclusion	Loss of data vs. low accuracy with misplaced electrodes	Electrode exclusion based on lesion sites and outlier power values
Brain regions	Cognition-related, difficulty of implantation	PFC, hippocampus, thalamus
LFP analysis toolbox	Reliability, validity, high prediction accuracy, robustness (few outlier values), broad range of metrics	EEGLAB, SIFT
Metrics	Partial lack of redundancy between metrics; mathematical and empirical validity; biological relevance and information; high accuracy	Multi-metric initial assessment and accuracy-based selection of optimal metric
Frequency bands (Hz)	Arbitrariness of borders, loss vs. redundancy of information	δ (1–4), θ (5–12), β (15–29), low-γ (30–49), mid-γ (51–99), high-γ (101–149)
Interval/episodes	Maximize training data, robustness and similarity of samples vs. pharmaco-kinetics/-dynamics	10 min intervals/20 s episodes
Train/test data split	Maximize training data, minimize outlier effects of test data	80/20 (∼8 mice/2 mice)
Cross-validation	High accuracy (trial-wise) vs. low over-fitting (mouse-wise)	Mouse-wise (emphasize over-fitting problem)
Hyper-parameters	Compute requirement and logistical effort vs. high accuracy Robustness of result	OPTUNA toolbox: max (test-accuracy) Use top-5 classifiers and average results
Classifier	High speed, high-accuracy, handling of outlier and missing values (electrodes), availability of efficient SHAP-calculation, low over-fitting	Light-GBM (also possible: XGB)
Normalization	Loss of information vs. over-fitting, data variability	Division by value from 10 min baseline
Feature extraction	Usage of optimized state-of-the art method, reproducibility, comparability	Shapley additive explanations (SHAP), max-normalized

#### 2.3.1 Pre-processing

The data was preprocessed in MatLab^®^ (MathWorks, Inc., MA United States) using custom written scripts. Electrophysiological data was decimated to 1 kHz, the *locdetrend* function (1 s moving window with 0.5 s step size) of the *Chronux* toolbox (Bokil et al., 2010) was applied to remove slow drift oscillations, and the *CleanLine noise* function from *EEGLAB* (Delorme and Makeig, 2004) was applied to remove potential 50 Hz noise and its corresponding harmonics (100, 150, 200, 250 Hz).

#### 2.3.2 Activity and connectivity estimation

Activity and connectivity were estimated for non-overlapping intervals of 20 s. To improve comparability of estimated parameters across animals, all computed values were normalized to the average values from the 10 min baseline-recording before drug application to control for absolute differences in signal strength between animals, sessions, and electrodes. Power-spectral density (PSD) as a measure for *activity* was estimated in the frequency range of 1–200 Hz with a resolution of 0.05 Hz using the multi-taper method provided by the *mtspecgramc* function of the *Chronux* toolbox. Vector auto-regressive (VAR)-based connectivity was calculated in the frequency range of 1–200 Hz with a resolution of 1 Hz using the *source information functional connectivity toolbox* (SIFT) of *EEGLAB* (Delorme and Makeig, 2004; Mullen, 2010; Delorme et al., 2011). Here, we calculated *coherence* (Coh) and *imaginary coherence* (iCoh) as metrics of non-directed connectivity, *Geweke-Granger causality* (GGC) (Geweke, 1982; Bressler et al., 2007) to represent *bivariate* directed connectivity, as well as *generalized partial directed coherence* [GPDC (Baccala et al., 2007)] and short-time direct directed transfer function [SdDTF or dDTF08 (Korzeniewska et al., 2008)] representing multivariate directed connectivity with normalization to the total outflow of each sending node or total inflow to each receiving node, respectively. Exact formulae and theoretical descriptions of these metrics can be found in (Mullen, 2010). A model-order of 30 and a sampling rate of 1 kHz were used for GGC calculation implying estimates in 30 ms segments. To generate activity and connectivity values as features for machine-learning, the values obtained with 0.5 Hz frequency resolution for each metric were averaged across common frequency bands: delta (δ, 1–4 Hz), theta (θ, 5–12 Hz), beta (β, 15–29 Hz), low gamma (low γ, 30–49 Hz), mid gamma (mid γ, 51–99 Hz), high gamma (high γ, 101–149 Hz). We spared 50, 100 and 150 Hz to avoid any potentially remaining contamination from grid noise and its harmonics.

To quantify the magnitude and direction of drug-induced changes (Figures 5B, E) the difference of the z-scored log2-transformed baseline-normalized amplitudes for each metric recorded under compound and vehicle was calculated:

$$\text{z}-\text{score}[\text{log}2({\text{A}}_{\text{C}}/{\text{A}}_{\text{BL}\_\text{C}})-\text{log}2({\text{A}}_{\text{V}}/{\text{A}}_{\text{BL}\_\text{V}})]$$

Whereby A_c_ is the amplitude of a metric recorded after compound; A_BL_C_ is the amplitude of that metric in the corresponding baseline before drug application (first 10 min); A_V_ is the amplitude of a metric recorded after vehicle; A_BL_V_ is the amplitude of that metric in the corresponding baseline before vehicle application. The log2-transformation has been applied to every individual baseline-normalized value (i.e., for every 20 s interval) to remove the distortion introduced by the baseline-normalization (ratio) that increases are numerically larger than equally-sized decreases, which would confound the difference metric. The z-score, calculated after pooling all data from across mice and the one compound and its vehicle, has been applied to adjust for different variability across connections and frequency bands (i.e., metrics). These values have been averaged across the last four intervals (minute 11–51) for Figure 5B and further analysis.

#### 2.3.3 Machine learning and SHAP-estimation

##### 2.3.3.1 Feature vectors

Feature vectors for every 20 s interval contained activity or connectivity parameters for each region or connection, respectively, in six frequency bands. In total, this amounted to 30 activity features (6 frequency bands * five regions; PSD) and 120 connectivity features per directed metric (6 frequency bands * 20 connections for GGC, dDTF08, GPDC) or 60 connectivity features (6 frequency bands * 10 connections) per non-directed metric (coherence, iCoh). In a first set of classifiers (main Figures 3A, B) all features were combined across metrics (510 features in total); in a second set of classifiers, decoding was done based on individual metrics at a time (feature numbers as stated above; Figures 3C, 6, 8A), and in a third set of classifiers (Figures 3D, 4, 5; only used for PSD and GGC), decoding was done based on features of one metric and one frequency band only (feature numbers equating to 1/6 of what is stated above) in order to avoid that more predictive frequency bands partially obscure the detection of features in other frequency bands with somewhat smaller predictive power; we term this set of classifiers and analysis “*frequency-subspace*” throughout and it is supposed to improve the detection of drug-induced changes across all frequency bands. The rationale for the latter is that frequency bands that are less reliably affected than others would not be highlighted by the classifiers as containing predictive information if more robustly changed features are available for the decoding; by forcing the algorithm to only use features from one frequency band at a time, the information that is available in that frequency band gets detected. To subsequently allow to compare across both frequency bands and compounds, the obtained and max-normalized SHAP values in these analyses were then scaled by the actually achieved accuracy of that classifier by multiplication with the term (accuracy-0.5); this ensures that even high SHAP-values get scaled down towards 0 if the accuracy of the classifier they derive from is approaching chance level (0.5).

##### 2.3.3.2 Temporal segmentation

Binary classification discriminating two pharmacological conditions, i.e., a compound against its vehicle or against another compound, based on feature vectors containing activity or connectivity parameters with balanced datasets was done using custom-written scripts in Python (Figure 1A). To accommodate for potentially distinct changes induced in early vs. late (steady-state) phases after a compound application—due to pharmaco-kinetic or -dynamic time-dependent processes—we separated the 60 min recording time in ten time-bins, which were treated as independent datasets during classification. Within each 10 min episode, the intervals of 20 s were used to calculate individual feature parameters; the 20 s lengths was chosen as it appeared to deliver marginally higher accuracy values compared to shorter or longer intervals in a pilot analyses. Therefore, for each classifier (for a 10 min episode), every mouse provided 30 20 s intervals, i.e., instances. Note, however, that a *post hoc* analysis using the final decoding approach with hyperparameter optimization revealed that there is no consistent difference in accuracy, between time bins of 10, 20 or 40 s, and only marginal decreases at shorter (5 s) and longer (75 s) periods in some cases (Supplementary Figure S1). Generally, interval length should be chosen as an optimal compromise between improving robustness of estimated parameters (provided by longer intervals) and increasing the amount of training data (enabled by shorter intervals). In alignment with a relative stability of accuracy values across the last four episodes (minute 11–50) after drug application, the accuracy and SHAP-values (see below) obtained from these intervals were averaged to obtain the final results. I.e., in the determination of drug-induced neurophysiological changes, the first 10 min after the injection were excluded from the final analysis given that both accuracy and SHAP-values largely deviated from the values obtained in the remaining 40 min.

##### 2.3.3.3 Choice of ML algorithm and determination of feature importance

Given the logistic and ethical limitations of pharmaco-electrophysiological experiments in animals, their output is usually bound to produce a relatively small number of instances, typically on the order of a few hundred. This limits the application of deep-learning algorithms—which typically rely on larger datasets—and favors the use of efficient classical approaches of classification, which include tree-based classifiers but also support vector machines (SVM), linear discriminant analysis (LDA), or general linear regression models (GLM). From a theoretical perspective, advanced tree-based classifiers appear superior for classification of electrophysiological data given that they are not only poised to deliver high accuracy, and provide many more parameters for hyper-parameter optimization (see below), but also because they can efficiently handle feature vectors with missing features (NaN values); given that some electrodes usually need to be excluded due to misplacement or corruption of the signal in electrophysiological datasets, the ability to deal with incomplete feature vectors is critical in order to avoid exclusion of whole subjects just because individual electrodes are unusable (this is also an important ethical consideration as the maximum exploitation of the data obtainable from each experimental subject is imperative). Furthermore, for the determination of the predictive power of each parameter of neural activity or connectivity (i.e., of each feature)—as a quantitative compound measure of the trial-to-trial reliability and amplitude of the drug-induced effect on that parameter—the *Shapley additive explanations* (*SHAP*)-method has been used, since it has been argued that it is the currently most advanced approach of interpretable ML (Lundberg and Lee, 2017; Lundberg et al., 2019; Lundberg et al., 2020; Molnar et al., 2024). SHAP-value extraction, however, is currently only implemented efficiently for *tree*-based classifiers using the *TreeExplainer* in Python (Lundberg et al., 2020; shap. TreeExplainer—SHAP latest documentation, n. d.). Although an alternative tool exists for other types of classifiers, the computing time is significantly longer. Finally, in pilot analyses, we compared SVM and several advanced tree-based approaches (Random-Forest; bagging, Dtree, AdaBoost, CatBoost, XG-Boost (XGB) (Chen and Guestrin, 2016) and *Light Gradient Boosting Machine* (LGBM) (Ke et al., 2017) finding, qualitatively, that XGB and LGBM mostly produced superior accuracies (not shown). All of the considerations above let us to focus on XGB and LGBM for the final analysis. Among these two algorithms, LGBM is several times (approximately 5–10x) faster than XGB in the case of our data, and did not appear inferior in accuracy (see Figure 6). At the level of SHAP-based extraction of predictive features, LGBM and XGB delivered highly correlated results (see Figure 6), suggesting that the additional use of XGB—although advertised for smaller datasets, compared to LGBM—is obsolete, and we conducted all our main analysis solely with LGBM. It is important to note, that the superior speed (without loss of accuracy) afforded by LGBM and the *TreeExplainer* is crucial to enable a multiplexing of analysis at several other levels that all help to improve the reliability of the outcome: a) An extensive hyper-parameter optimization - in our case, requiring to compute 50,000 classifiers per individual analysis (i.e., per metric, drug and potentially frequency band; see below). b) The calculation of multiple classifiers (500 in our case) from more than one set of hyperparameters to improve robustness of results by later averaging of accuracy and SHAP-values. c) The application of the frequency-subspace method described above—requiring a sixfold increase in the number of classifiers to be calculated at the level of both hyper-parameter optimization (6 * 50,000) and final analysis (6 * 500) to detect drug-induced changes in frequency bands which are affected as well, albeit with lower reliability. d) Assessment of multiple metrics of connectivity (Strahnen et al., 2021a). e) Further desired validation, e.g., by comparing mouse-wise with trial-wise cross-validation or by cross-validation across time intervals (both not performed here). Therefore, in real-world applications of interpretable ML to extract drug-induced neurophysiological changes, computational efficiency—as provided by LGBM and the TreeExplainer—appears essential to enable high analytical quality and reliability of results.

##### 2.3.3.4 Cross-validation, hyperparameter optimization, and final ML analysis

Decoding (prediction) accuracy was estimated for each individual classifier from prediction attempts on data from two pseudo-randomly chosen mice that have not contributed to the training data (*out-of-the-sample* or *mouse-wise cross-validation*), which ensured that the final result is biologically robust and reliable. This choice corresponded to an 80:20 split between training and testing data; i.e., for almost all drugs (except amphetamine) individual classifiers were trained on the data from eight mice (8 * 30 = 240 instances per 10 min episode) and prediction accuracy was tested on a further 2 * 30 = 60 instances. For amphetamine, two mice did not respond to the compound (no locomotor increase after drug application) and were excluded from the analysis to avoid contamination of the results with non-typical data; here training data was contributed by six mice. For each classifier a new split between training and test data was done, ensuring that every mouse contributed sometimes to the training and sometimes to the test data, with an equal distribution across mice.

Hyperparameter optimization was used to ensure high accuracy and low levels of overfitting—the former ensures that the algorithm captures the predictive features (i.e., drug-induced changes) optimally (high compliance), the latter implies that accuracy is not based on the learning of regularities that are not due to the effect of the compound but due to other extrinsic factors. The use of mouse-wise cross validation with multiple repetitions across which the identity of mice contributing to the training vs. the test dataset was pseudo-randomized ensured that high accuracy could not be attained by over-fitting. Therefore, maximum test accuracy has been set as the sole optimization goal. Hyperparameters were optimized in the *OPTUNA* toolbox (Akiba et al., 2019) (Preferred Networks, Inc.), which performs an intelligent search through a non-discretized hyper-parameter space, requiring a considerably smaller number of assessed hyper-parameter combinations while allowing the exploration of a wider range of hyperparameter-values compared to classical grid-search approaches with complete permutations of all hyperparameter-combinations with a fixed range and discrete step-size of each hyperparameter. This efficiency is crucial to enable hyper-parameter optimization for every new set of classifiers, e.g., when multi-plexing the analysis approach with the frequency-subspace method (see above). As for the final analysis, cross-subject prediction with mouse-wise cross-validation was performed during hyper-parameter optimization with 10 classifiers being calculated for each combination of hyperparameters (but with a different split of mice across training and test data, see above). 5,000 hyperparameter sets were allowed to be explored for every optimization (requiring 50,000 classifiers to be calculated in total) and the top 5, according to test accuracy, were used for the final analysis.

In that final analysis, from which actual accuracy and SHAP-values were determined, 100 iterations were calculated for each of the top 5 hyperparameter sets - again, each with mice being pseudo-randomly assigned to training and test data. The accuracy values were averaged across these five sets for every iteration, yielding a population of 100 accuracy values—each derived as an average of five classifiers calculated from the same underlying training/test-data split with different hyperparameter sets—across which statistical analysis (as shown in Figure 3) was performed. That use of the top 5 hyperparameter sets (as opposed to just the best *one*) and high number of 100 iterations for each set, in addition to the separate calculation and averaging across four 10 min intervals of expected steady-state pharmacological activity served to ensure the best possible reliability and robustness of the final result. The absolute values from the resulting SHAP-values from each of these 5 * 100 classifiers were max-normalized between 0–1, weighted by the accuracy of the individual classifier they were derived from applying a multiplication by (accuracy-0.5), and averaged across the 500 classifiers and, for the ultimate analysis of steady-state changes (Figures 5A, 7), across the four last time intervals of the recording.

Subsequent statistical analysis was done in SPSS (IBM, NY, United States) as described in Results. All custom-written analysis scripts for *MATLAB* and *Python* can be found on GitHub (https://github.com/KaetzelLab/DA-Pharmacology-ephys-analysis-2024.git), all raw data (down-sampled to 1 kHz) on https://doi.org/10.12751/g-node.w7s6wv.

## 3 Results

### 3.1 Dopaminergic compounds induce distinct behavioural and physiological activity

To assess drug-induced connectivity, mice were injected with a dopaminergic compound after 10 min of habituation and baseline recording in a novel open field, followed by 50 min of further open-field habituation and recording. Behaviourally, compared to trials with vehicle application, an increase of locomotor activity was seen after injection of amphetamine, whereas activity decreased when mice were injected with clozapine, raclopride, or SCH23390 (Figures 1B,C).

**FIGURE 2:**
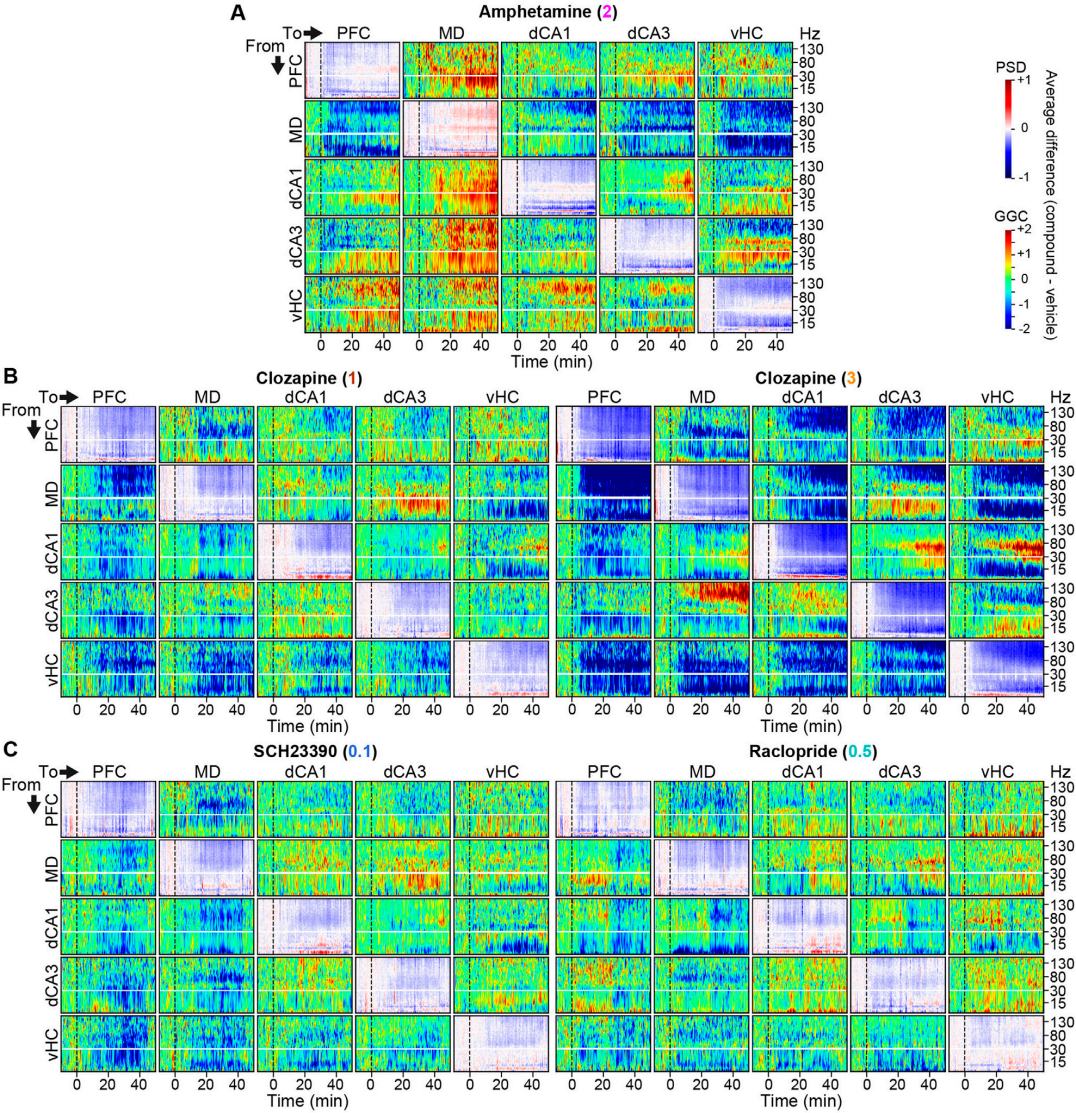
Connectivity and activity spectrograms of dopaminergic compounds **(A–C)** Spectrograms showing average numeric difference between log2-transformed baseline-normalized GGC-spectrograms recorded under 2 mg/kg amphetamine **(A)**, 1 [**(B)**, left] or 3 [**(B)**, right] mg/kg clozapine, 0.1 mg/kg SCH23390 [**(C)**, left], or 0.5 mg/kg raclopride [**(C)**, right], and those recorded under vehicle (rainbow-scale). Panels along the diagonal lines represent corresponding power-spectral density (PSD) difference spectrograms (blue-red-scale). Vertical black lines indicate time of injection. Horizontal white lines indicate 30 Hz and break the plot along the y-axis to improve visibility of changes in lower frequencies. Note the similarity between the two doses of clozapine.

In order to evaluate potential neurophysiological effects of each compound qualitatively, we calculated difference-spectrograms, whereby the average log-transformed and baseline-normalized directed connectivity (measured with GGC) and activity (PSD) before and after injection of each compound was plotted after subtraction of the same values for the corresponding vehicle sessions. After compound applications, multiple changes were observed in average activity and directed GGC connectivity between the recorded brain regions (Figures 2A–C). For example, qualitatively, amphetamine increased the communication from all recorded regions *to* the MD-thalamus in the β-γ frequency range while decreasing the connectivity *from* the MD in the high-γ and δ frequency bands. Amphetamine also enhanced *local* activity in the low-γ range, but decreased it in most lower frequency bands, as described previously for its derivative DOI (Rangel-Barajas et al., 2017). Generally—and as expected—a majority of connections and frequency bands appeared to be affected by the applied compounds (Figures 2A–C), raising the necessity of an approach to extract the most pronounced and robust compound-induced changes.

### 3.2 Decoding of compound identity from activity and connectivity

**FIGURE 3:**
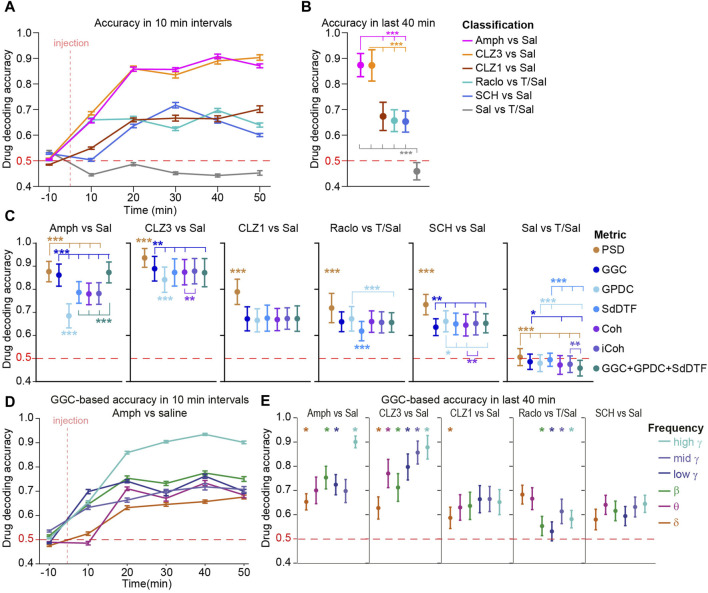
Decoding of drug identity from neural activity and connectivity **(A)** Decoding accuracies of LGBM algorithms trained to predict the compound a mouse has received (vs. vehicle) based on five connectivity and one activity metric *combined* plotted in 10 min intervals. Note that classifiers trained to discriminate vehicle-types perform at chance level (50%) **(B)** Same data as **(A)** but averaged across the last four intervals (steady-state action of compounds) **(C)** Same analysis as in **(B)** but using classifiers that were trained on features from only *one* metric, indicated by colour **(D)** Similar analysis as in **(A)** for amphetamine vs. saline, but classifiers were trained on features from only one metric (GGC) and frequency band (indicated by colour according to legend in (E)) **(E)** Similar analysis as in **(C)** just for classifiers that were trained on features from only one metric (GGC) and frequency band (as shown in **(D)**. See Supplementary Figure S2 for the equivalent analysis based on PSD. Error bars represents s. e.m. in time series data **(A, D)** and S.D. in aggregate data **(B, C, E)**. Asterisks indicate pairwise **(B)** or paired **(C, E)** Sidak *post hoc* tests conducted after significant main effects in one-way **(B)** or repeated-measures **(C, E)** ANOVA; in **(B, C)** the number of asterisks represents the lowest significance level of all indicated comparisons; in **(C)** only metrics that differ from *all* other metrics are indicated by asterisks without additional comparison lines. *p < 0.05, **p < 0.01, ***p < 0.001; in **(E)** only frequency bands that are distinct from all other at *p* < 0.05 (or higher significance level) are indicated, for clarity. No statistics was applied in **(A)** and **(D)**. Horizontal red dashed lines represent chance level.

Therefore, we deployed interpretable machine-learning (ML) (Strahnen et al., 2021b; Molnar et al., 2024) using predictive power in single-trial data to extract those changes by binary classifications between data recorded after injection of each compound from data recorded under vehicle (Figure 1A). Cross-validated decoding accuracies above chance level (50%, given that the datasets were always balanced) indicated that a compound induced consistent neurophysiological alterations, whereby higher accuracies scale with the robustness of such changes. Usage of *single-trial* (as opposed to average) data and *mouse-wise cross-validation*—whereby data that is used to measure the decoding accuracy of the algorithm is taken from mice that contributed *no* data to the training dataset—constitute high and conservative demands for the decoder ensuring that only robust changes, occurring stably across time and subjects are detected. In a first set of classifiers, we combined five different metrics of local *activity* (PSD) and *directed* (GGC, SdDTF, GPDC) and *non-directed* (Coh, iCoh) *connectivity* in six frequency bands (see Methods) as a single predictive vector to provide the maximum available information obtainable from LFP data. Classifiers were trained on 20 s data snippets from 10 min intervals. Whereas decoding accuracies remained around chance level (0.5) in the baseline interval before injection, they rose for all compounds in the intervals following injection (Figure 3A). As a negative control, decoders attempting to discriminate the two vehicle types remained around chance-level throughout (Figure 3A). Average prediction accuracies after the wash-in phase (20–50 min post-injection) reached just below 90% for amphetamine and the higher dose of clozapine, and around 65% for raclopride, SCH23390 and the lower dose of clozapine, indicating effective decoding of robust neurophysiological alterations induced by every drug (Figures 3A, B).

To assess, if changes affected rather activity within regions or connectivity between regions, we computed a second set of classifiers for which predictors derived only from one metric. Somewhat surprisingly, predictors trained with the information on local activity (power) had marginally, but significantly, higher decoding accuracies compared to classifiers trained on any of the connectivity metrics, for all tested compounds (Figure 3C), suggesting that local activity is affected more robustly by the drugs than connectivity. Among the connectivity metrics, GGC appeared marginally more informative than the other metrics as it allowed for significantly higher decoding accuracy in the discrimination of amphetamine and 3 mg/kg clozapine vs. vehicle (Figure 3C). This suggests that GGC is the most suitable of the tested connectivity metrics for these datasets, and it was used for all subsequent analysis.

Next, we investigated if communication was affected by a given compound only in particular frequency bands by calculating a third set of classifiers based on GGC parameters from single frequency bands at a time (*frequency-subspace classifiers*). Astonishingly, all frequency bands carried information about the presence of almost every one of the tested compounds, to some degree. Mostly however, communication was affected more robustly in certain frequency bands compared to others; e.g., the high-γ (100–150 Hz) band provided a higher decoding accuracy than any other frequency for amphetamine and the 3 mg/kg clozapine, whereas raclopride affected δ and θ more robustly than any other frequency band (Figure 3D). A similar pattern of frequency-specificity was observed for local activity (Supplementary Figure S2).

Given the rich information contained in all frequency bands and the partly very focused effect of compounds in small frequency ranges observable in some spectrograms (Figure 2), we investigated whether a more fine-grained slicing of the frequency-space would yield higher accuracy as information contained in different sub-regions of classical frequency bands becomes more detectable for the classifier. Notably, for example, the dopaminergic agonist apomorphine was shown to shift the peak of striatal gamma-frequency oscillations from 50 to 80 Hz (Valencia et al., 2012), which would go possibly undetected with our current frequency-bands. Therefore, we calculated classifiers akin to those shown in Figure 3C for PSD and GGC albeit with frequency-intervals of 2 Hz from 1 to 30 Hz and intervals of 10 Hz from 31 to 150 Hz, leading to 28 instead of six frequency bands. In line with the broader pharmacological effects in the frequency space of connectivity, this alteration did either not lead to higher accuracy or even mildly decreased drug decoding accuracy (Supplementary Figure S3); for local activity (PSD), slight increases of accuracy were observed for amphetamine and CLZ1, but not in the remainder. This suggests that a different choice of frequency bands is unlikely to reveal prominent pharmacological effects that go beyond those captured by the six frequency bands analysed in this study.

### 3.3 Compound-induced changes of neural activity and connectivity

Whereas the frequency-subspace analysis revealed that dopaminergic compounds affect distinct frequency bands more than others, a more fine-grained extraction of predictive power of individual connectivity and activity parameters is necessary to map anatomically specific alterations. We used the SHAP method (Lundberg and Lee, 2017; Lundberg et al., 2019; Lundberg et al., 2020), which combines several advantages of older techniques of interpretable ML (Molnar et al., 2024) to extract the most robust drug-induced physiological changes. In order to improve the reliability of the obtained results, we extracted SHAP-values from 500 classifiers per compound, metric and frequency-subspace, and averaged them after max-normalization and weighting with the classifiers’ cross-validated accuracy (see Methods).

**FIGURE 4:**
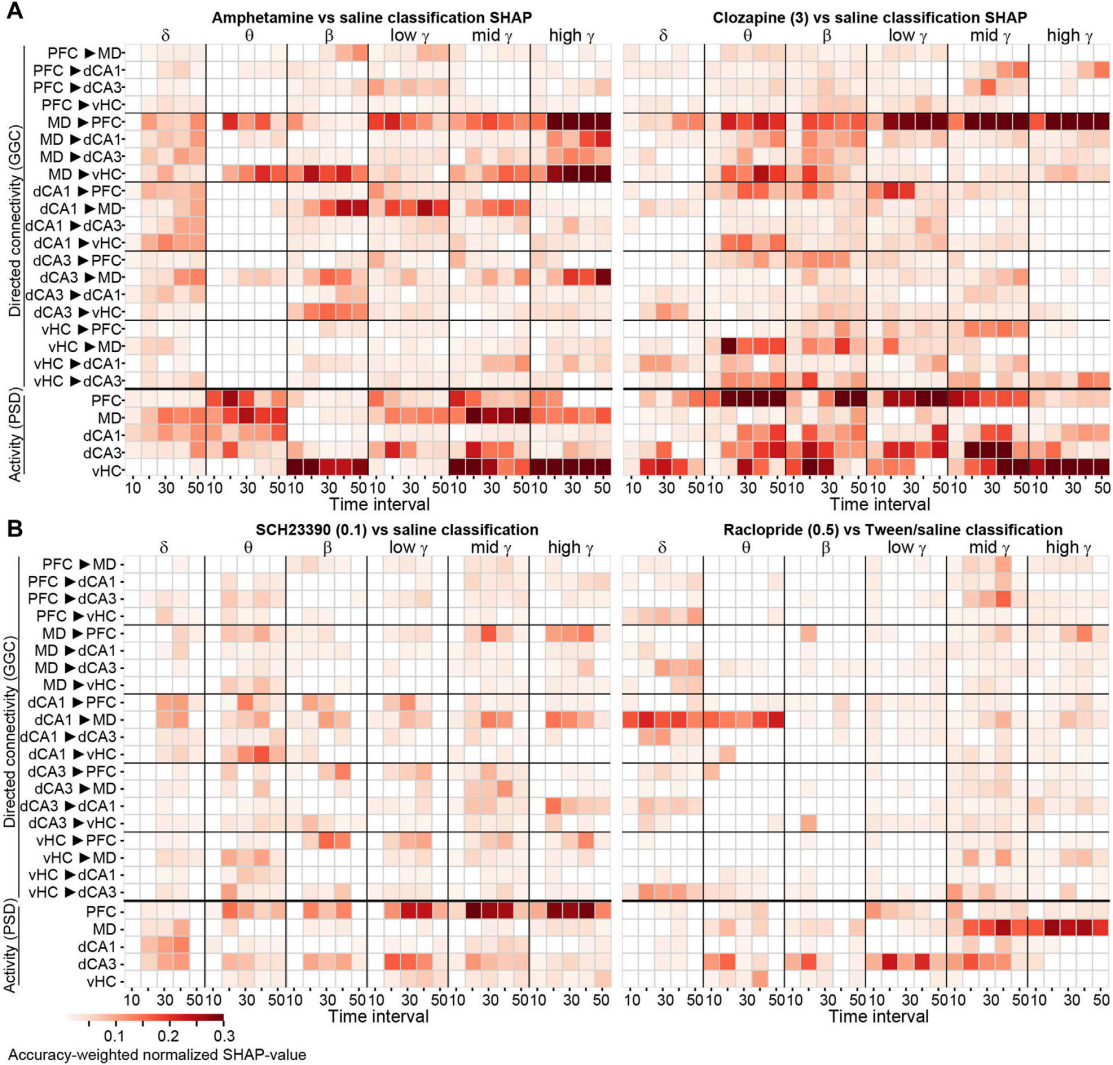
SHAP-based feature importance obtained with frequency-subspace decoding displayed over time **(A, B)** Accuracy-weighted average max-normalized SHAP-values obtained with the frequency-subspace method for binary classifications of 2 mg/kg amphetamine [**(A)**, left] and 3 mg/kg clozapine [**(A)**, right], 0.1 mg/kg SCH23390 (B, left) and 0.5 mg/kg raclopride [**(B)**, right], each against vehicle, displayed in 10 min intervals post-injection for each frequency band (x-axis) and connection or region (y-axis). Note that–although shown together–classifiers were calculated separately for GGC-based connectivity (top) and PSD-based activity (bottom). Features with normalized average SHAP-values <0.01 are shown as white.

We found that strong SHAP-values were often repeatedly detected for the same predictive connectivity or activity parameters in distinct datasets representing consecutive time intervals (Figures 4A, B), which underlines the validity of this approach to detect real compound-induced physiological changes that persist across time. Qualitatively, deviations from reoccurring patterns affected mostly the first interval, which likely reflects differences in the early effects a compound exerts during wash-in compared to stable effects in the steady state, or simply the absence of the latter during the first minutes after application (see, e.g., connectivity changes in δ and θ-bands induced by either compound only at later time points; Figures 4A, B).

**FIGURE 5:**
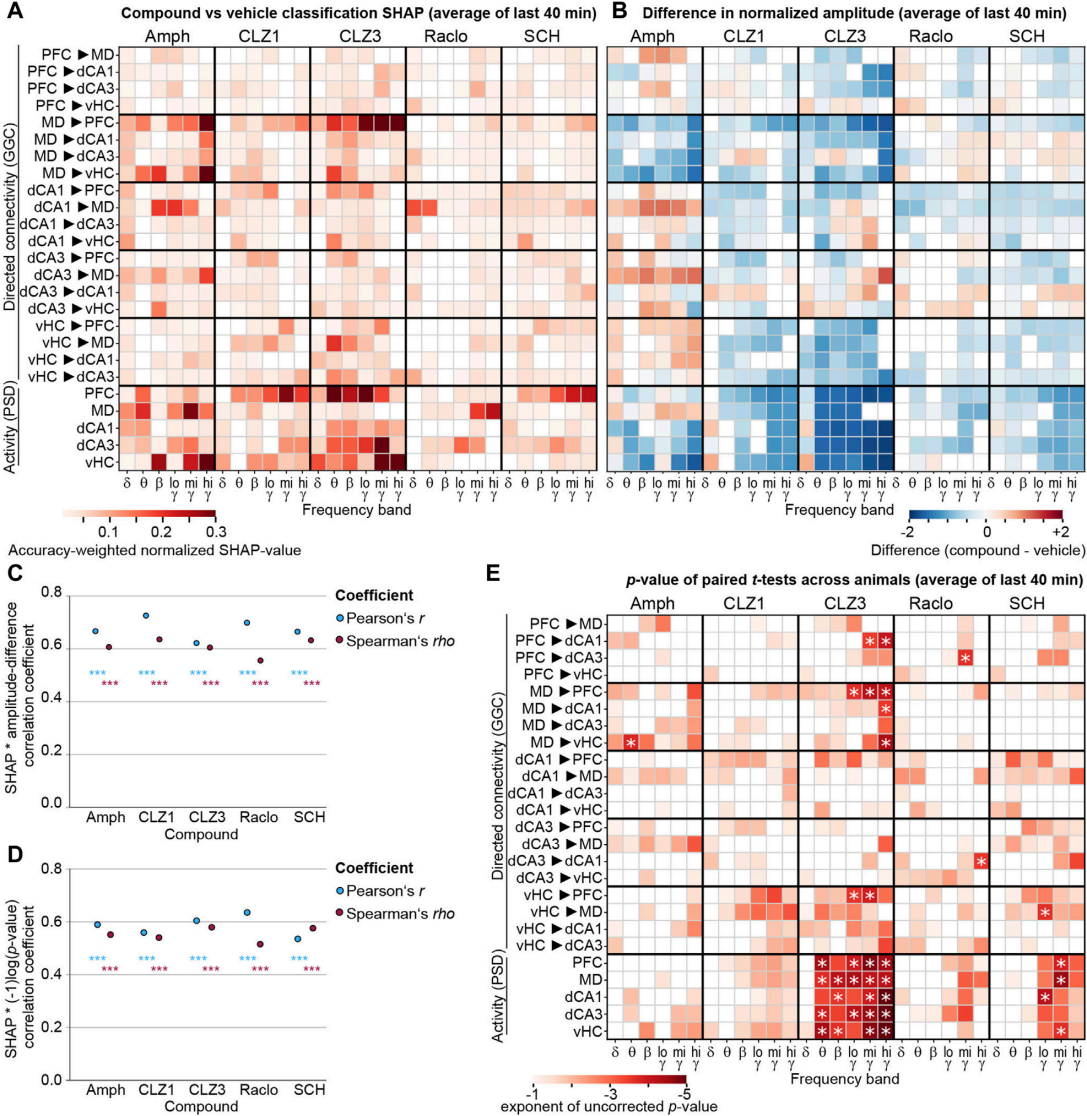
Compound-induced changes of neural activity and connectivity **(A)** Same analysis as in Figure 4, but SHAP-values have been averaged across the last four time-intervals (covering effects occurring 11–50 min post-injection) for all five tested compounds and six frequency bands (x-axis). See Supplementary Figure S4 for SHAP-results obtained *without* the frequency-subspace method, for comparison **(B)** Average numeric difference of the z-score of log2-transformed amplitudes of a given parameter under compound and vehicle, as a measure of the *size* of the changes; averaged across the last four time-intervals. The z-score was calculated across all mice and all sessions from one compound and its vehicle pooled to adjust for different variability between connections and frequency bands (i.e., metrics). Blue and red colours indicate decreases and increases, respectively, compared to vehicle, for metrics of connectivity (top) or activity (bottom). In panels **(A, B)**, parameters for which the accuracy-weighted average normalized SHAP values was <0.01 are shown as white **(C)** For the average over the last four intervals (representing minutes 11–50 post-injection), the primary measure of *robustness* of compound-induced changes [accuracy-weighted normalized SHAP-value from frequency-subspace classifiers; as shown in **(A)**] was correlated to the absolute value of the primary measure of the *size* of the compound-induced changes (the difference of the z-scored log2-transformed baseline-normalized amplitudes for each metric recorded under compound and vehicle; as shown in (B)); both without thresholding. The information whether the alteration was an increase or decrease of activity compared to vehicle is, hence, not considered for the correlation. Pearson correlations have been computed to take into account the actual size of SHAP- and difference measures, Spearman correlations have been computed to only regard relative sizes (order number). Correlations have been done for vectors which contained all GGC- and PSD-metrics of all frequency bands in a linearized order, for every compound (x-axis) **(D)** Similar to **(C)**, but correlations between SHAP-values (as shown in **(A)**, but without thresholding) and absolute values of log-transformed *p*-values (as shown in **(E)**, but without thresholding) **(E)** *p*-values for paired *t*-tests comparing within-subject averages of z-scored log2-transformed and baseline-normalized connectivity or activity values [as used for the difference calculation in **(B)**] with averaging done across all 20s-intervals from minute 11–50 post-injection. Uncorrected *p*-values <0.1 are shown in colour, white indicates *p* ≥ 0.1; singular asterisks indicate significance after Bonferroni-adjustment for multiple comparisons (*p* < 0.05/150). ***p < 10^−10^ for all correlations.

To aggregate the robust steady-state changes, we averaged the SHAP-values across the last four time-intervals for all tested compounds, representing the essential analysis outcome of our approach detecting robust drug-induced neurophysiological changes with interpretable ML (Figure 5A). Due to the accuracy-based weighting of SHAP-values obtained in frequency-subspace classifiers, the displayed values can be compared across compounds—with amphetamine and 3 mg/kg clozapine showing the most pronounced changes. Notably, particularly robust changes were sometimes not very frequency-specific; for example, both doses of clozapine affected MD→PFC connectivity and PFC activity robustly across virtually all frequency bands (Figure 5A).

In order to determine the numeric size and direction of the detected neurophysiological alterations induced by a given compound compared to vehicle, we plotted the arithmetic difference between the average z-scored and log-transformed normalized value of every neurophysiological parameter obtained after injection of drug and that measured after vehicle (Figure 5B). The linearized vectors of the absolute values of these difference metrics correlated significantly with the linearized vectors of SHAP-values for every compound (*p* < 10^−12^ for both Spearman and Pearson correlations; Figure 5C), validating that our interpretable ML-approach detects changes that are not only robust but also strong in actual amplitude. All anti-dopaminergic drugs had in common that they reduced local activity in all measured regions in almost all frequency bands (except for δ), similar to what was observed with olanzapine (but not some other second generation antipsychotics) before (Dimpfel, 2007). Likewise, most connectivity - with some notable exceptions in hippocampal connections - was decreased by anti-dopaminergic compounds (Figure 5B).

For methodical comparison, we also followed the classical statistical approach of calculating paired *t*-tests across within-animal averages for all metrics. The resulting patterns of *p*-values correlated with the SHAP-value patterns obtained by the ML-approach (*p* < 10^−10^; Figure 5D), but the individual *p*-values mostly did not survive Bonferroni correction for multiple comparisons (*p* < 0.05/150 metrics; Figure 5E). This suggests that the ML-approach is largely able to identify effects that would also be obtained by frequentist statistics but is superior in terms of sensitivity *to* and quantification *of* drug-induced effects.

**FIGURE 6:**
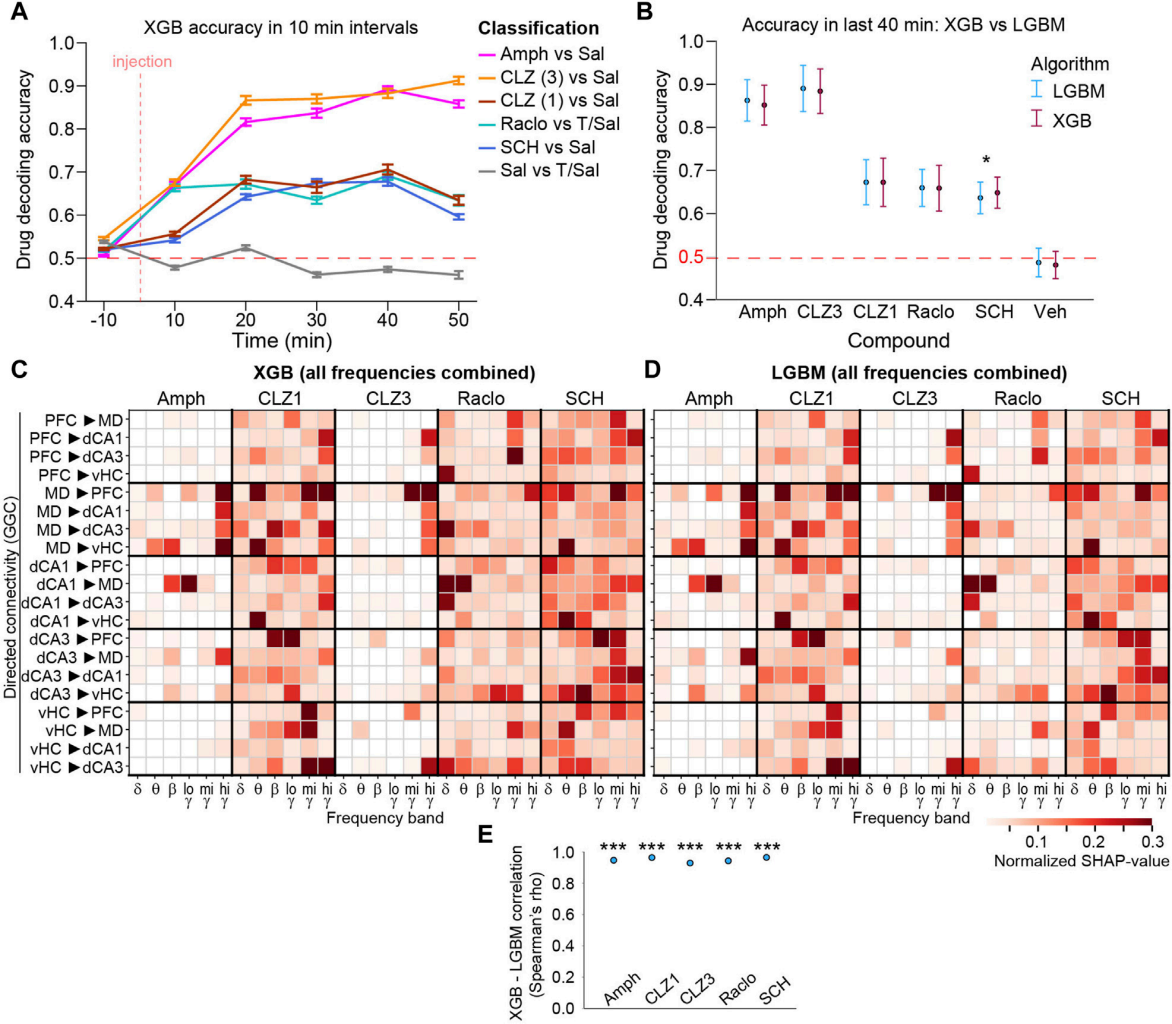
Validation of LGBM results using the XGBoost (XGB) algorithm **(A)** Decoding accuracy for binary drug-vs-vehicle discrimination calculated in 10 min intervals from GGC directed connectivity. Error bars, s. e.m. **(B)** Average decoding accuracy in last four intervals (minute 11–50 post-injection) for LGBM and XGB algorithms based on GGC. Asterisk indicates significant difference (*p* < 0.05; one-way ANOVA). Error bars, S.D. **(C, D)** Average normalized SHAP values from last four intervals from XGB **(C)** and LGBM **(D)** classifiers trained with GGC connectivity from all frequencies combined. Note that classifiers have not been calculated with the frequency-subspace method due to the slower computation time of XGB, and are hence, not accuracy-weighted). In both cases, SHAP values are derived as averages from 100 classifiers each from the top 5 hyperparameter sets **(E)** Spearman’s *rho* from correlations of the linearized SHAP-value vectors (as shown in C-D) obtained with XGB and LGBM, calculated within each of the compounds. ***p < 10^−52^ for all correlations. Features with normalized average SHAP-values <0.01 are shown as white.

To further validate these results, we repeated the same GGC-based ML analysis with the XGB algorithm (Chen and Guestrin, 2016) and detected very similar patterns of changes that were significantly correlated to those revealed with LGBM-classifiers for all compounds (Spearman’s *rho* > 0.92, *p* < 10^−52^; Figures 6A–E).

### 3.4 Compound-specific alterations are prominent in specific connections

**FIGURE 7:**
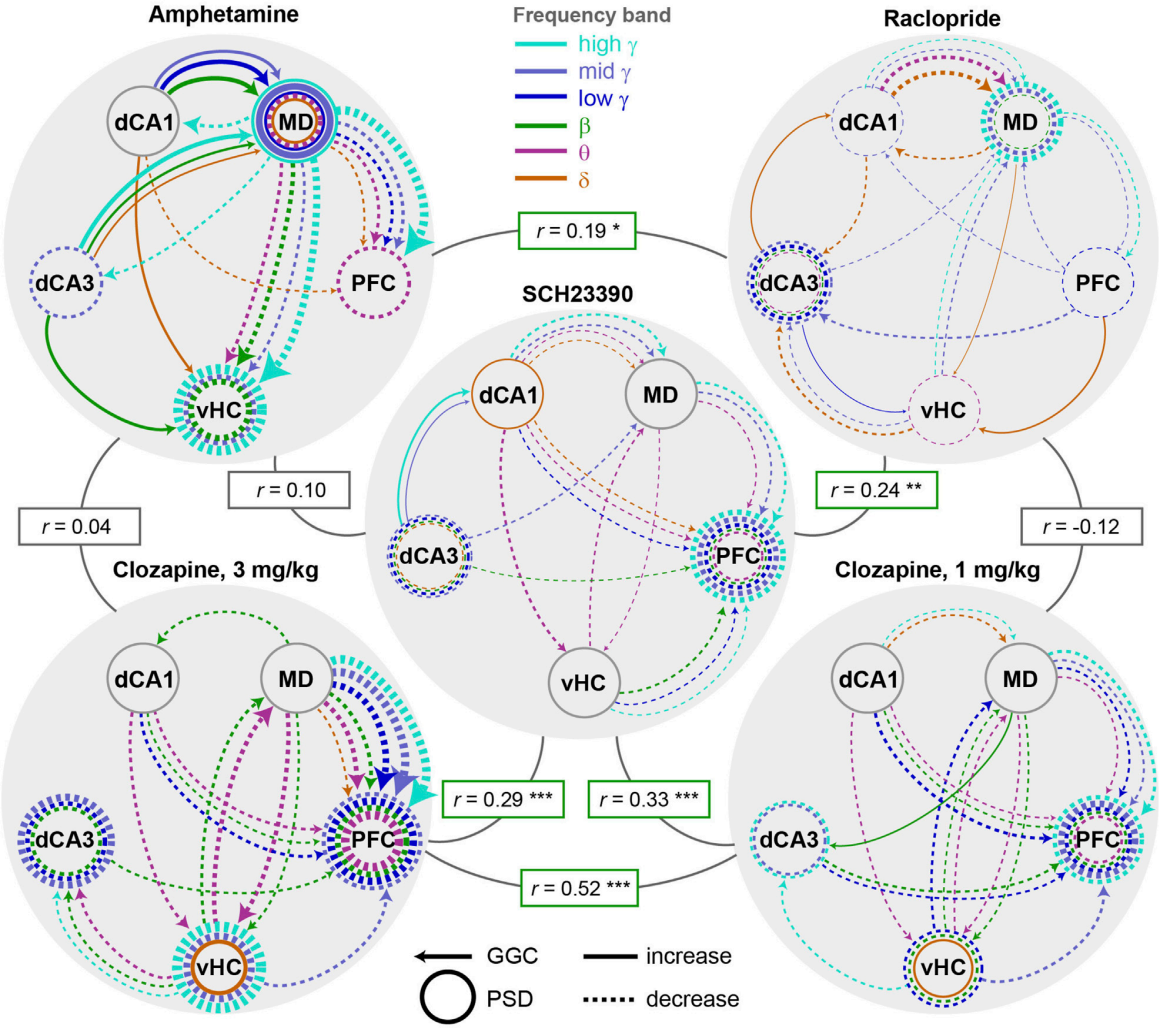
Compound-specificity of changes to neural activity and connectivity **(A)** Depiction of the top 20 (out of 120) connectivity (arrows, GGC) and top 10 (out of 30) activity (circles, PSD) parameters as ranked by the accuracy-weighted SHAP-values (Figure 5A). Frequency-bands are colour-coded; dashed and solid lines represent decreases and increases (determined from amplitude difference values shown in Figure 5B), respectively, induced by a given compound relative to its vehicle. Line thickness represents 10 * SHAP-value [pt]. Spearman-correlations between the pattern of all 150 neurophysiological variables are indicated in rectangular boxes. Non-indicated Spearman’s *rho* for amphetamine x 1 mg/kg clozapine equals 0.065 (n.s.), and for raclopride x 3 mg/kg clozapine equals −0.125 (n.s.), *p* > 0.1). *p < 0.05, **p < 0.01, ***p < 0.001.

In order to obtain a clearer view on the drug-specific neurophysiological changes, we generated graphs depicting the top 20 connectivity and top 10 activity parameters as ranked by the accuracy-weighted normalized SHAP-value (Figure 5A). Among those most prominent changes, almost all effects induced by anti-dopaminergic drugs were *decreases* of connectivity and activity (Figure 7A). A notable exception to this, was a re-configuration in the δ-band communication induced by raclopride which entailed increases (MD/PFC→vHC; dCA3→dCA1) as well as decreases (vHC/dCA1→dCA3; dCA1↔MD). Generally, multiple compound-specific dominant alterations emerged. For example, amphetamine disproportionately impacted especially MD-thalamic *connectivity* and *activity*, whereas clozapine affected mainly prefrontal *activity* and thalamic *connectivity*. Hence, the majority of the most prominent alterations caused by amphetamine were not opposing, but, in fact, often surprisingly similar to those induced by anti psychotics—clozapine in particular—entailing, especially, strong decreases in MD→PFC/vHC connectivity across most frequency bands. In fact, *all* tested compounds prominently decreased MD→PFC γ-band communication. In contrast to antipsychotics, however, amphetamine strongly *increased* dCA1/dCA3→MD connectivity and local MD activity across multiple frequency bands, which—among the most robust alterations in the sampled circuit—appeared to be the main distinction between the pro- and anti-dopaminergic compounds. All anti-dopaminergic compounds, in turn, had in common that they decreased dCA3 and PFC γ-power. To quantify similarities and differences, we calculated bivariate Spearman correlations between the SHAP-value patterns of the 150 electrophysiological parameters (Figure 7A). Reassuring the validity of our approach, the two doses of clozapine showed the highest correlation of their neurophysiological alterations (*rho* = 0.52; *p* < 10^−11^) and shared 21 (70%) of the 30 parameters that were changed most robustly (out of 150). Surprisingly, they also correlated strongly with SCH23390 (*rho* > 0.28; *p* < 10^−3^), but hardly with raclopride (*rho* < 0; *p* > 0.5). This suggests that—in the cortico-thalamo-hippocampal network we surveyed—D1-antagonism creates a larger share of the connectivity changes observed under clozapine compared to its D2-antagonism, in line with a relatively broader expression of the corresponding gene *Drd1* compared to *Drd2* in murine neocortex and hippocampus (Supplementary Figure S5) (Hodge et al., 2019). At the level of frequency bands, clozapine and SCH23390 mainly influenced the θ−β−γ range, whereas effects in the δ- and, specifically, mid-γ-band dominated the effects of raclopride.

### 3.5 Discrimination between compounds confirms specific circuit alterations

**FIGURE 8:**
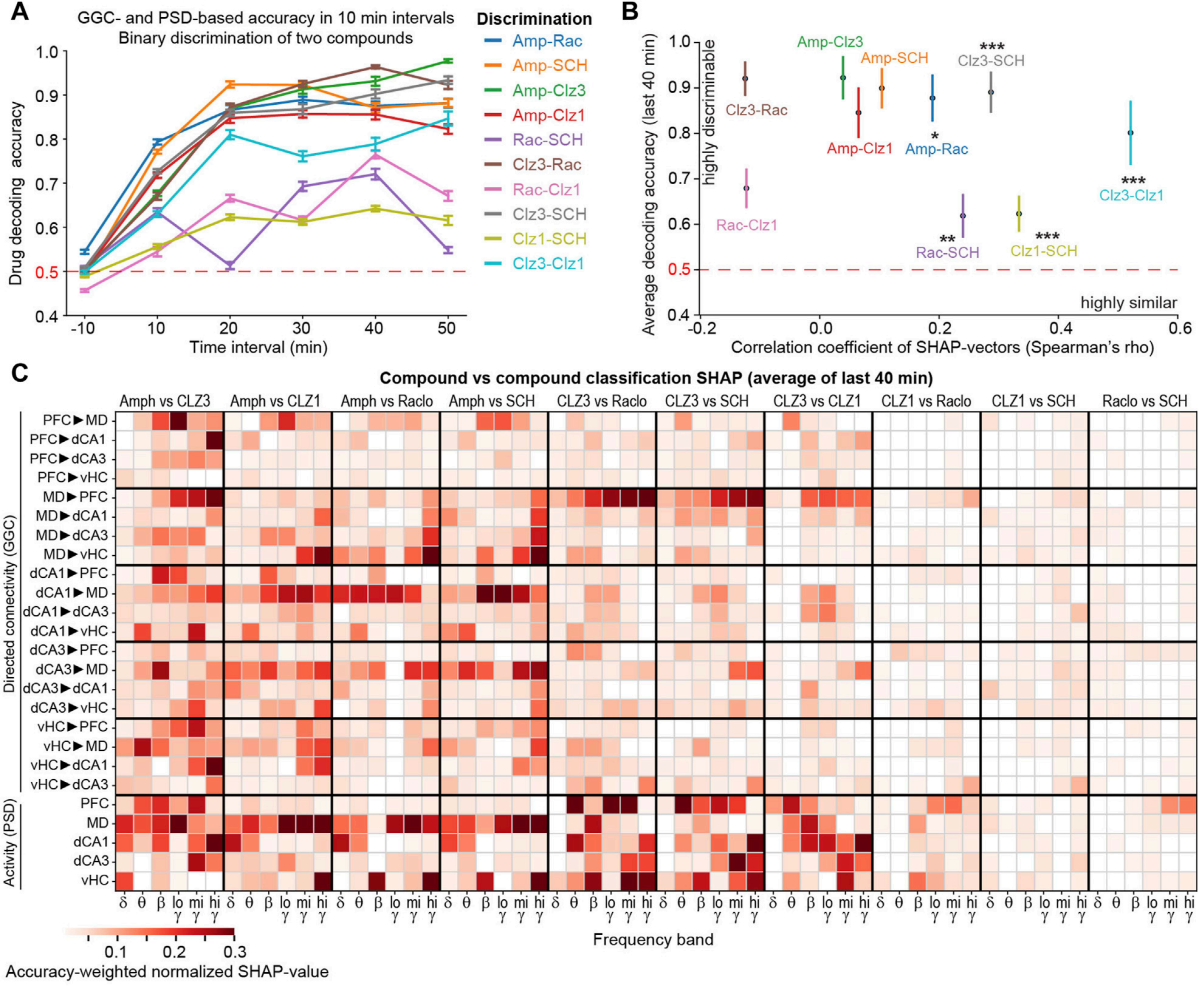
Binary classification between distinct compounds **(A)** LGBM decoding accuracies for pairwise discrimination of two compounds based on GGC and PSD combined over time in 10 min intervals. No statistics applied **(B)** Average decoding accuracy in the last four intervals (as shown in (A)) plotted against Spearman’s *rho* coefficients of bivariate correlations between the average normalized SHAP-value patterns of the same pair of compounds. Asterisks indicate significance level of *rho*. Error bars represent s. e.m. in **(A)** and s.d. in **(B)**. **p < 0.01, ***p < 0.001 **(C)** Same analysis as in Figure 5A, just for binary classifications between two compounds (as shown in panel (A)); SHAP-values have been averaged across the last four time-intervals (covering effects occurring 11–50 min post-injection) for all 10 possible comparisons between tested compounds and six frequency bands (x-axis).

To further evaluate the differences and similarities of the drug-induced connectivity patterns, we trained binary classifiers to discriminate within all possible pairs of applied compounds based on GGC and PSD parameters of all frequencies combined. Highest decoding accuracies were achieved when discriminating amphetamine or the high dose of clozapine against other drugs (Figure 8A). When plotting such accuracies (indicating how discriminable two compounds are) against correlation coefficients of compound-vs.-saline SHAP-value patterns (indicating how similar drug-induced changes are in terms of robustness) an expected inverse relationship became apparent for all instances that involved amphetamine or 3 mg/kg clozapine (Figure 8B), i.e., the two compounds with the strongest changes (Figure 3): among those pairs, the two doses of clozapine showed the lowest discriminability and the highest similarity, followed by clozapine vs. SCH with a higher degree of difference, whereas the remainder showed low positive or even negative correlations but could be discriminated with high average accuracies above 80% (Figure 8B). However, combinations that involved *only* compounds with weaker effects (raclopride, SCH, 1 mg/kg clozapine) broke that pattern and average discriminability remained low, irrespective of the correlation. This also led to the counter-intuitive result that the two doses of clozapine were easier to discriminate than the low dose of clozapine from raclopride and SCH23390. These effects are likely due to the overall weaker robustness of the alterations they induced and a certain similarity between effects of dopaminergic antagonists (Figure 8B).

To identify the most prominent differences between drug classes, we extracted SHAP-value patterns from the binary classifiers (analogous to Figure 5A). These analyses confirmed our previous results (Figures 5A, 7A); indicating, for example, that amphetamine distinguished itself from all anti-dopaminergic drugs by an increase in output from dorsal hippocampus to thalamus and in thalamic activity which both occurred broadly in frequency-space (Figure 8C). It also revealed that the high dose of clozapine was most distinguishable from the other anti-dopaminergic compounds by a broad change in MD→PFC connectivity and PFC activity as well as in hippocampal β−γ-power (Figure 8C). We further confirmed such prominent differences using multi-class classification between all four compounds (Supplementary Figure S6). Overall, these analyses confirm that every dopaminergic compound induces a unique and complex pattern of neurophysiological changes.

### 3.6 Drug-induced connectivity patterns are not driven by changes in locomotion

**FIGURE 9:**
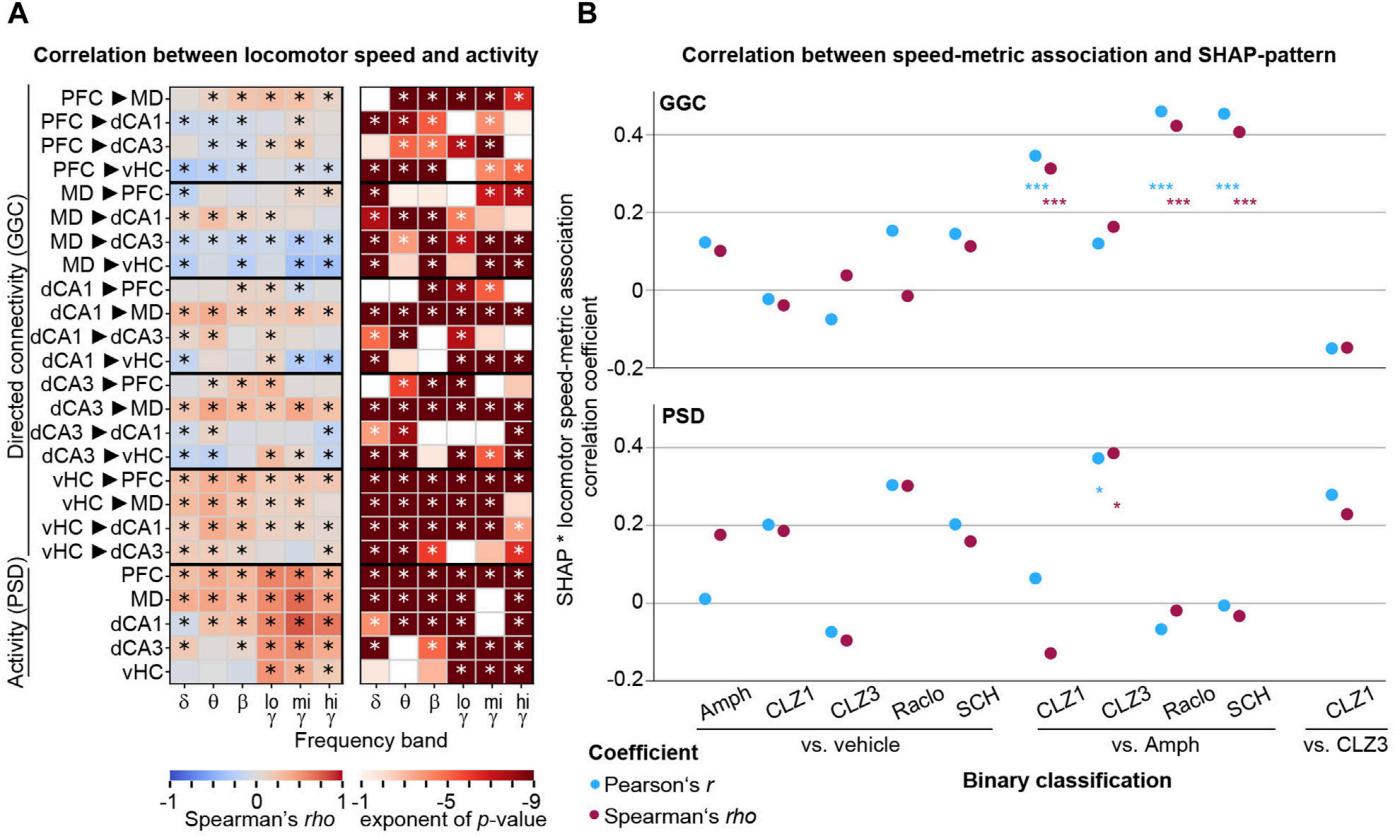
Correlations between neurophysiological parameters and locomotor activity **(A)** Spearman correlations (*rho*, left; *p*-value, right) between connectivity (GGC) or activity (PSD) parameters (y-axis) across frequency bands (x-axis) and locomotion speed. *p*-values <0.1 are shown in colour in the right panel, and asterisks indicate significance with Bonferroni-adjustment (*p* < 0.05/150) **(B)** Coefficients of bivariate correlations between SHAP-value patterns for drug-discriminations as shown in Figures 5A, 8C and patterns of associations between neurophysiology and locomotor speed [absolute value of the correlation coefficient shown in **(A)**], shown for GGC-connectivity (top) and PSD-activity (bottom) for the binary discriminations indicated on the x-axis.

An important caveat of all analyses of physiological effects of neuropharmacological compounds that alter exploratory locomotor activity - as is the case for dopaminergic drugs (Figures 1B, C) - is the unresolved causal relation between such behavioural activity and neural oscillations. It is well established that dorsal hippocampal theta oscillations correlate with locomotor speed; in this case, however—using optogenetic modulation of oscillations—it has been determined that neurophysiological activity controls that behaviour (Bender et al., 2015), not the reverse. In such cases, locomotor behaviour is not a confounding factor because the causal chain goes from compound to physiology to behaviour. In contrast, a confound can be assumed in the opposite case, i.e., if locomotor speed causally changes a neurophysiological variable which in itself is not affected in the same way directly by the drug. Given that we find surprisingly different changes to connectivity induced by the anti-dopaminergic compounds that all depress locomotor activity to a similar degree (Raclo, SCH, CLZ1; Figure 1C), it is unlikely that our results are largely determined by the same factor of decreased locomotor activity. Nevertheless, we explored this option further and, firstly, correlated baseline-normalized locomotor activity in 20 s episodes from minute 11–50 post-injection of the two vehicle sessions (*N* = 3,960) to the baseline-normalized value of every metric in such episodes as it was used for the ML-analysis. We found that the correlation between locomotor speed and neurophysiological metrics extended far beyond theta oscillations in dHC; in fact, 90 of the 120 GGC connectivity parameters (−0.38 < *rho* < 0.42) and 26 out of 30 activity parameters (−0.08 < *rho* < 0.78) correlated significantly with spontaneous locomotor activity fluctuations in untreated animals (Spearman’s *rho*; corrected *p* < 0.05/150; Figure 9A). We next correlated the resulting patterns of correlation coefficients with SHAP-value patterns of binary drug-vs-vehicle and drug-vs-drug classifications (Figures 5A, 8C) to estimate what the maximal potential confound by locomotion would be. Generally, correlations were low and mostly non-significant, with some exceptions of discriminations of amphetamine against dopaminergic antagonists (Figure 9B). This suggests that—at least for the results obtained for drug-vs-vehicle classification—the identified connectivity changes are not caused simply by changes in locomotor speed.

## 4 Discussion

We here presented an approach combining multi-site LFP recordings and state-of-the-art interpretable machine learning to extract neurophysiological effects of psychoactive compounds. The recording of multiple sites, initial assessment of multiple connectivity metrics, and the reliance on single-trial predictive power to extract robust effects aids in reducing the *street-light effect* in pharmaco-electrophysiological studies. We applied multiple measures to ensure the robustness of the obtained result, including cross-subject prediction, subject-wise cross-validation, efficient hyperparameter optimization and the use of multiple high-scoring hyperparameter sets, each contributing 100 classifiers with altered train:test-data splits, the frequency-subspace method to detect changes also in less affected frequency bands, and the use of an advanced yet computationally highly effective, tree-based algorithm (LGBM) in combination with the *TreeExplainer* for advanced, SHAP-based extraction of predictive feature-weights. The identification of similar changes across two types of tree-based classifiers (LGBM and XGB, Figure 6), across two doses of the same drug (clozapine; Figures 5A, 7A), and across time-intervals within each of the compounds (Figures 4A, B) served as positive controls of the robustness of the approach, whereas the chance-level accuracy when discriminating against saline (Figures 3A, B) served as negative control. The accuracy-weighted normalized SHAP-value measuring the *predictive power* or *robustness* of drug-induced changes (Figures 5A, 7A, 8C) is suggested as a more reliable indicator for pharmacologically induced neurophysiological alterations than *p*-values (Figure 5E) for high-dimensional, low-*N* electrophysiological datasets; it correlated strongly with the (compound-vehicle) difference of the actual amplitudes of each baseline-normalized metric indicating the *size* of the induced changes and with patterns of *p*-values of *t*-test-based analysis of the same data (Figures 5C, D), which further confirmed the validity of the interpretable ML approach to extract drug-induced differences between physiological states. We expect that the approach presented here could help to bring previously requested standardization to the field of preclinical neuropsychopharmacology (Drinkenburg et al., 2016) and to extract more information from existing and future datasets in the field.

With respect to the specifically investigated dopaminergic compounds, we revealed patterns of changes of connectivity and activity induced by every compound, that exceeded previously reported changes in individual frequency bands of averaged *local activity* obtained with depth LFP- or scull EEG-electrodes in terms of complexity (Dimpfel, 2009). For example, it was shown before that the antipsychotics risperidone and clozapine decrease neocortical and hippocampal power across most frequencies, but especially in the γ-range (Jones et al., 2012; Ahnaou et al., 2016; Drinkenburg et al., 2016; Hudson et al., 2016; Ahnaou et al., 2017; Gener et al., 2019; Sun et al., 2021), which we confirm here for all dopaminergic antagonists, but particularly for clozapine [Figures 2B, 5A, B; note, however, that different patterns have been found with different antipsychotics, that further depend on time point and dose (Dimpfel et al., 1992; Sebban et al., 1999; Dimpfel, 2007; Dimpfel, 2009)]. Importantly though, our approach, reveals many further fine-grained details such as particularly strong and spectrally broad decreases of MD→PFC and bidirectional MD↔vHC communication by clozapine, of dCA1→MD→PFC and vHC→PFC communication by D1R-antagonism, and a complex re-configuration of δ-band connectivity by raclopride (Figure 7A), to just name a few. When comparing these changes to those induced by the pro-dopaminergic compound amphetamine, we found that some alterations—in particular a broad-band reduction of MD→PFC/vHC communication—were unexpectedly similar to those induced by clozapine, but that the key difference was a prominent enhancement of β−γ communication originating from the dorsal hippocampus and an increase of MD-thalamic local activity by amphetamine, which could be central to its opposing psychological effects. Whereas we cannot offer a mechanistic explanation for the partial similarities between amphetamine and anti-dopaminergic drugs or for the partial differences within the latter class, it is worthwhile noting that similar findings of differences between drugs of the same pharmacological class as well as strong similarities between drugs of distinct classes, have been described before (Dimpfel, 2007; Dimpfel, 2009; Ahnaou et al., 2014) and underline the complexity of neurophysiological actions of individual neuropsychiatric compounds. Despite the complexity, certain patterns emerged that were largely reproducible across time points and, in the case of clozapine, across drug-doses which overlapped by 70% in the most robustly changed frequency-specific neurophysiological parameters.

Despite this advancement, several limitations of both our analytical pipeline and our specific conclusions remain. With respect to the latter, given the low number of males in the dataset (2/10), we could not draw conclusions regarding sex-related similarities or differences of these pharmacological effects. Secondly, whereas our correlation analysis in Figure 9 renders it very unlikely that most of our SHAP-based results reflect neurophysiological changes induced by altered perceived locomotor speed, a confound by this factor cannot be completely ruled out. While this is a principal problem when analyzing neural effects of locomotion-altering compounds (not specific to our study or approach), the introduced ML-based analysis—in contrast to frequentist analysis using multivariate ANOVA or ANCOVA—cannot easily include locomotor speed as covariate to somewhat control for its influence. Thirdly, the analysis approach presented here for local activity could be further improved by including aperiodic components of the electrophysiological signal, such as baseline shifts which can confound the measurement of amplitudes in frequency space (Valencia et al., 2012; Redondo et al., 2014; Donoghue et al., 2020). Fourth, in the light of previous studies, it needs to be noted that further complexity is expected to arise with an extension of dose range and recording time, given that local activity has been reported to change - partly very profoundly - across these dimensions after application of pro- and anti-dopaminergic drugs (Dimpfel, 2007; 2009). Furthermore, our chosen six frequency bands may not capture all oscillatory processes or may be too broad to detect shifts in the peak-frequency of oscillations [as seen with dopaminergic modulation before (Valencia et al., 2012)] and may therefore miss some neuropharmacological effects. Indeed, our spectrograms show that especially local activity displays more fine-grained frequency-specificity like a sharp decrease of the alpha-band power by amphetamine (Figure 1D) that was also observed with the dopaminergic agonist L-DOPA previously (Dimpfel, 2009). Therefore, our approach could be further advanced by introducing even more frequency bands, such as α or low- and high-β bands (Dimpfel, 2009). However, given the focus of our study on *connectivity*, where changes appeared less fine-grained along the frequency axis and difficult to subdivide by classical frequency-boundaries (Figures 2A–C), we did not follow this approach here. In line with this observation, our exploratory analysis with fine-grained frequency bands (Supplementary Figure S3) did not yield higher decoding accuracy for connectivity-based classification, but showed some minor improvement for activity-based decoding in the case of two compounds. A data-driven—rather than *a priori*—subdivision of frequency bands may be considered in future to optimize accuracy and the extraction of mechanistically interpretable neurophysiological changes.

Overall, this study showed that the application of interpretable ML to large sets of activity and connectivity data obtained by multi-site depth recordings may reveal distinct effects of psychoactive compounds, which mostly occur preferentially—but not exclusively—in certain frequency bands, regions, and connections.

## Data Availability

The datasets presented in this study can be found in an online repository. The name of the repository and DOI can be found below: https://doi.org/10.12751/g-node.w7s6wv.
