# Supplementary material for: Neural effects of dopaminergic compounds revealed by multi-site electrophysiology and interpretable machine-learning
Source: Front Pharmacol. 2024 Jul 9;15:1412725. doi: 10.3389/fphar.2024.1412725 (PMC11263031; doi:10.3389/fphar.2024.1412725)
Supplement: Supplementary file 1 [file DataSheet1.pdf]

Supplementary Information

Neural effects of dopaminergic compounds revealed by multi-site electrophysiology and interpretable machine-learning

Sampath K.T. Kapanaiah [1], Holger Rosenbrock [2], Bastian Hengerer [2], Dennis Kätzel [1,*]

[1]Institute of Applied Physiology, Ulm University, Ulm, 89081 Ulm Germany

[2]Boehringer Ingelheim Pharma GmbH & Co. KG, Div. Research Germany, Biberach an der Riss, Germany

* Correspondence: dennis.kaetzel@uni-ulm.de; +49 731 500 33770; Fax +49 731 500 33779; Institute of Applied Physiology, Ulm University, Albert-Einstein-Allee 11, 89081 Ulm, Germany

Supplementary Methods

Surgery

Electrode implantation surgeries were performed under general isoflurane-anaesthesia (approx. 1.5% for maintenance) supplemented by a broad peri-operative analgesic regime including local anaesthesia with bupivacaine (2 mg/kg; 8 μl/g injection volume), and general analgesia with buprenorphine (0.1 mg/kg; 10 μl/g injection volume) and meloxicam (5 mg/kg; 10 μl/g injection volume), similarly as previously described (Strahnen et al., 2021). A heating pad (Harvard Apparatus, MA, US) controlled by a temperature sensor was used maintain the animals body temperature and ointment (Bepanten™ , Bayer, G) was applied to the eyes to prevent drying. Single polyimide-insulated tungsten wires of 50 µm diameter (WireTronic Inc., CA, US) were implanted in the right hemisphere, with reference to Bregma (in mm), into the PFC (dual electrode at AP +1.8-1.9, ML 0.3-0.35; 1.8-1.9 and 1.0-1.4 below pia), MD (AP -1.2, ML 0.3, 2.7-2.9 below pia), dCA1 (AP -1.9-2.0, ML 1.5, 1.4 below pia), dCA3 (AP − 1.8, ML 2.0-2.1, 1.8 below pia) and vHC (dual electrode at AP -3.1-3.2, ML 2.9-3.0, 3.3-3.4 and 3.8-3.9 mm below pia). In the case of dual electrodes, the data from the electrode that was placed most optimally in the target region (centered in PrL or at the vHC fissure, respectively) was used for analysis whereas the data from the second electrode was not used. Stainless steel screws (1.2 mm diameter, Precision Technologies, GB) were implanted in the contralateral hemisphere ca. 1 mm from the midline above the cerebellum (AP -5.5) for ground and reference, and above the auditory cortex (A1) to measure auditory evoked potentials in separate experiments (not described here); both screws were connected with a 120 µm PTFE-insulated stainless-steel wire (Advent Research Materials Ltd., UK). Finally, an additional stainless-steel wire was placed above the contralateral temporal cortex (AP -2.0, ML -1.0) as an additional source of a reference signal (not used in the current analysis). All electrode wires were connected to pins in a dual-row 10-pin connector (Mill-Max, GB).

Electrophysiological recordings

Animals were tethered to enable electrophysiology recordings and then placed into a fresh clear type III plastic cage (length 43 cm, width 22 cm, height 20 cm; Tecniplast, IT) containing clean sawdust. Animals were allowed to explore for 10 min while recording the pre-injection baseline activity; subsequently, compound or vehicle were injected i.p. and the mice were immediately back into the same open field to record neural activity for another 50 min. The animals' movement in the open field was video-tracked with ANY-maze (Stoelting, US), and the distance travelled was calculated in 10 min time bins.

Prior to testing, a 32-channel RHD2132 headstage for amplification and digitization (Intan Technologies, CA, US) was plugged into the implanted connector via a custom-built adaptor that interfaced a 36-pin Omnetics connector (A79022–001, MSA components, DE) with another 10-pin Mill-Max connector. The adaptor was wired so that all signals were referenced to the ground signal obtained from above the contralateral cerebellum. The headstage was wired with a light-weight SPI cable (Intan Technologies) to a custom-made motorized Open-MAC commutator (Kapanaiah and Kätzel, 2023) (now available from Labmaker, DE, and Cambridge NeuroTech, GB) from which a further SPI-cable was connected to an Open-Ephys (Siegle et al., 2017) acquisition board (https://open-ephys.org, USA; obtained through the Open-EPhys store at Champalimaud, Portugal) via two light-weight flexible SPI-cables (Intan Technologies). Data was recorded in the Open-EPhys acquisition software (Siegle et al., 2017) at a sampling rate of 20 kHz and simultaneously online band-pass filtered at 0.1–300 Hz (for all remaining analysis of LFP signals).

Electrolytic lesioning and histological processing

After all experiments had been conducted in this cohort, electrode placements *post-mortem,* electrolytic lesions were made after breathing ceased under terminal ketamine/medetomidine anaesthesia. Immediately afterwards, animals were transcardially perfused with PBS followed by 4% paraformaldehyde (PFA)/PBS and brains were post-fixed for 24 h in PFA/PBS. Coronal sections of 60 µm were cut on a vibratome in PBS and then washed 3 times in PBS, stained with DAPI, and mounted for inspection of lesion sites on an epifluorescence microscope (DM6, Leica, G).

Supplementary Figures

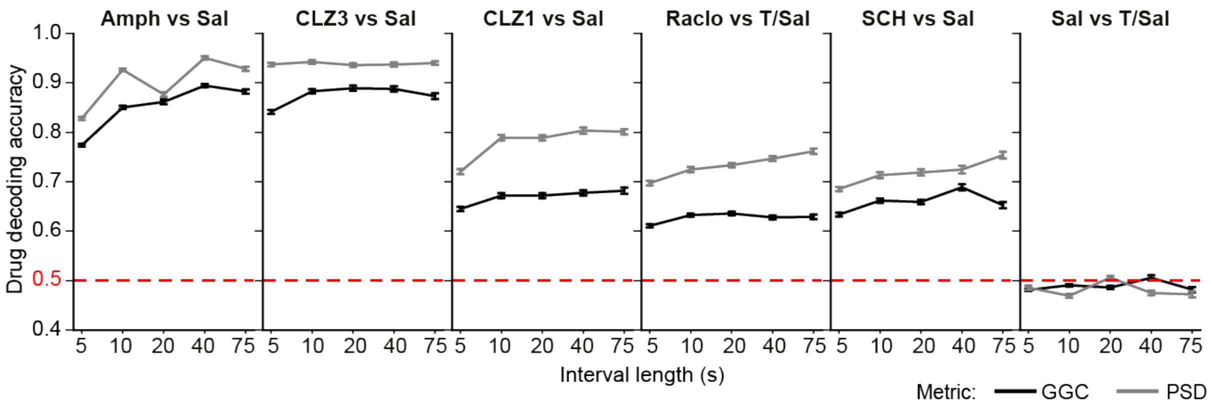

Supplementary Figure 1. Dependency of decoding accuracy on interval length. Average decoding accuracy for binary compound-vs-saline classifications (compounds labelled by colour) using local activity (PSD) or directed connectivity (GGC) plotted against the length of the intervals that constituted individual instances. Classifiers with optimized hyperparameters have been computed and tested like in Figure 3C (there shown for 20 s interval length), including hyperparameter optimization (5000 repetitions) and calculation of cross-validated decoding accuracy for 100 classifiers each of the top 5 hyperparameter sets, across which accuracy values were averaged. Accuracy values represent averages across the four last intervals and each datapoint represents mean±s.e.m. across such 100 averages. An inverted-U shape is expected given that very short intervals likely lead to larger variability across the population of values and very long intervals lead to lack of training data (fewer intervals), both of which are expected to decrease accuracy. No statistics applied.

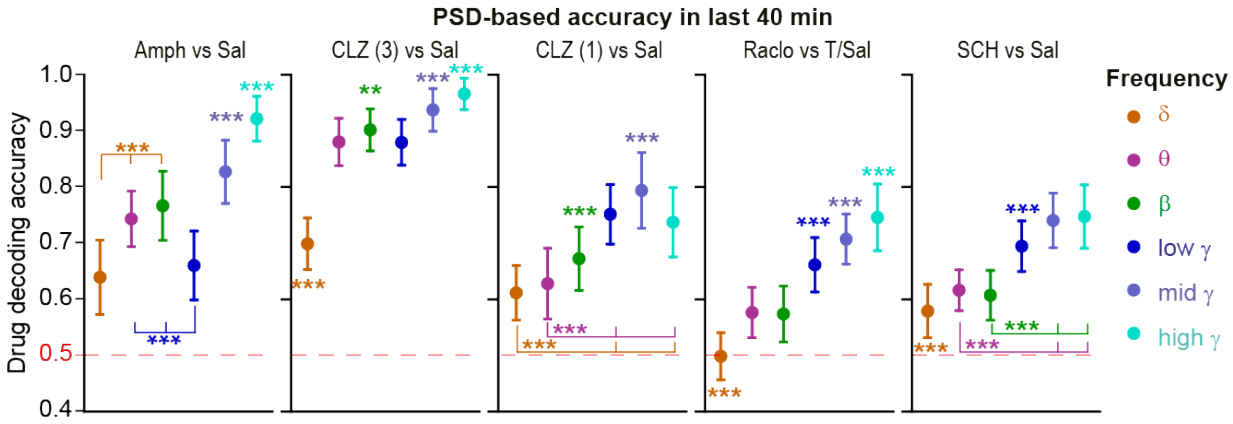

Supplementary Figure 2. Frequency-subspace decoding accuracy for PSD. Average compound decoding accuracy (vs. vehicle) across the last 4 intervals of recording (11-50 min post-injection) when using classifiers trained with local activity (PSD) parameters from only one frequency at a time (equivalent to main Figure 3E for connectivity). Error bars represent s.d. Asterisks indicate Sidak post-hoc tests conducted after significant main effects in repeated-measures ANOVA; the number of asterisks represents the lowest significance level of all indicated comparisons; metrics that differ from all other metrics are indicated by asterisks without additional comparison lines. * $P < 0.05$, ** $P < 0.01$, *** $P < 0.001$. Red dashed line represents chance level.

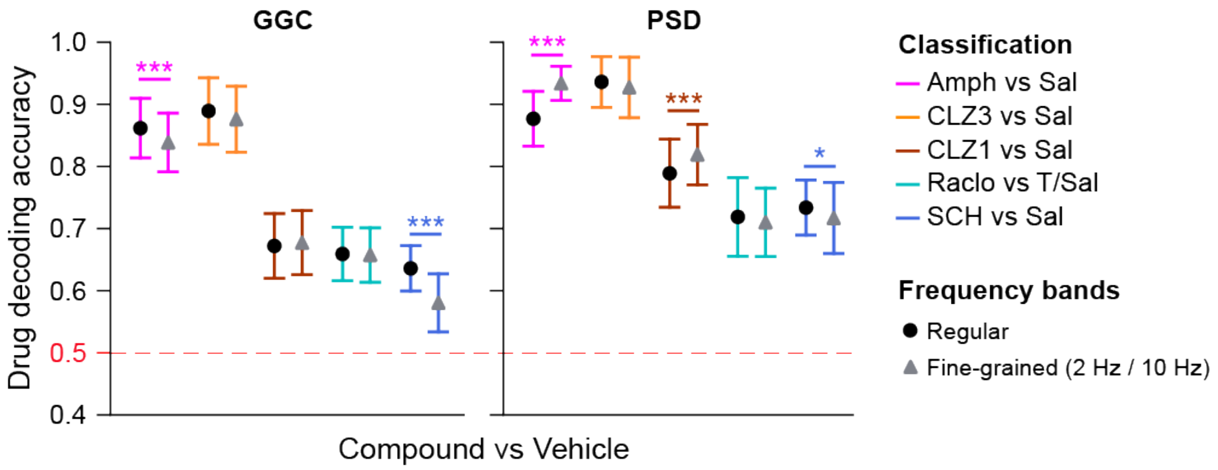

Supplementary Figure 3. Decoding accuracy with fine-grained frequency-distribution of features. To assess if the 6 frequency bands chosen for the analysis presented throughout the main manuscript (regular) are too broad to capture important drug-induced changes, we repeated the same ML analysis using 28 bands across the same 1-150 Hz range: bands of 2 Hz width from 1-30 Hz and bands of 10 Hz width from 31-150 Hz (fine-grained). Asterisks indicate paired *t*-tests; error bars indicate s.d. * $P < 0.05$, *** $P < 0.001$. Red dashed line represents chance level.

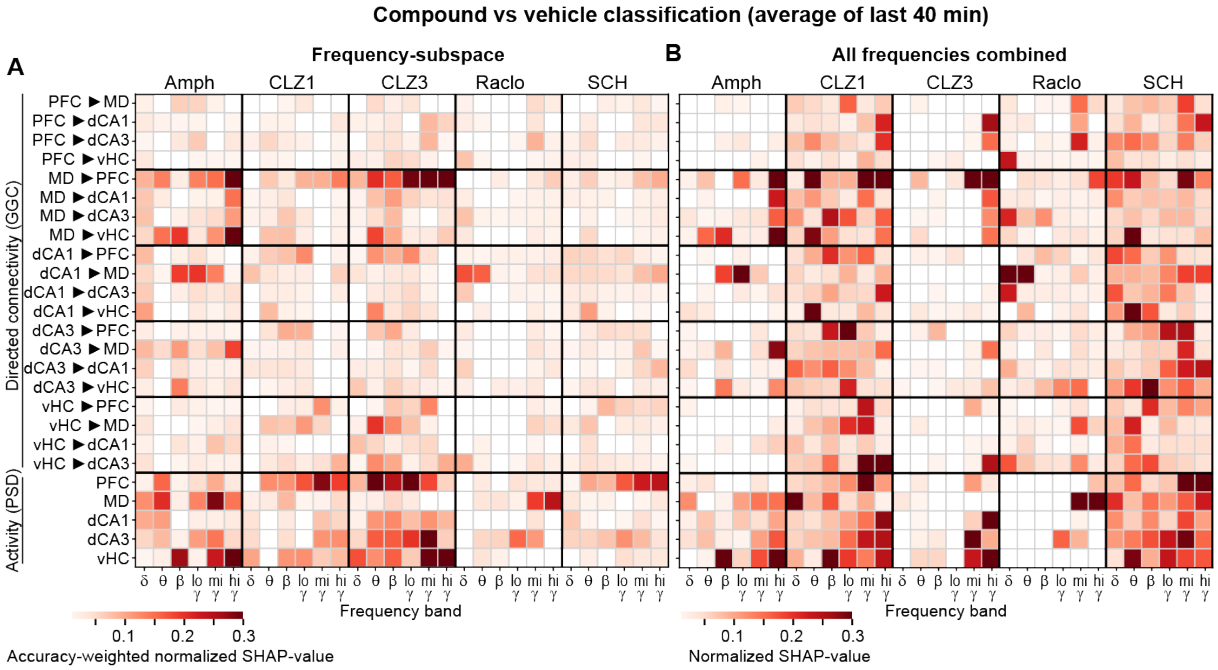

Supplementary Figure 4. SHAP-based feature importance obtained with and without frequency-subspace decoding. (**A-B**) Left is identical to main Figure 5A (shown here for comparison to (B); max-normalized SHAP-values obtained *with* (A) and *without* (B) the frequency-subspace method for binary classifications of 2 mg/kg amphetamine (Amph), 1 and 3 mg/kg clozapine (CLZ), 0.5 mg/kg raclopride (Raclo), and 0.1 mg/kg SCH23390 (SCH), each against vehicle, averaged across the last 4 intervals (representing minute 11-50 post-injection) displayed for each frequency band (x-axis) and connection or region. Note that – although shown together – classifiers are calculated separately for GGC-based connectivity (top) and PSD-based activity (bottom). Furthermore, SHAP-values shown for classifiers without frequency-subspace are not scaled by the classifier's accuracy (hence, relatively strong SHAP-values may occur despite relatively weak effects of compounds). Features with normalized average SHAP-values < 0.01 are shown as white.

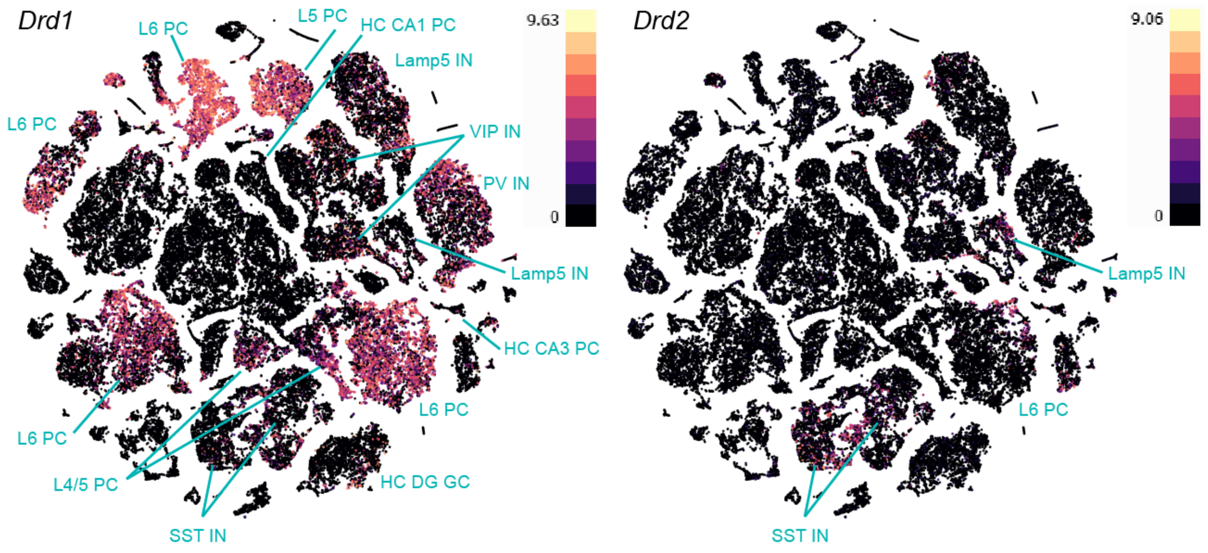

Supplementary Figure 5. Expression pattern of *Drd1* and *Drd2* across 20 neocortical and hippocampal areas. Single-cell RNA-sequencing based expression strength (coded by colour, sale to the top right of each subpanel, representing log2(expression count)) of *Drd1* (left) and *Drd2* (right) across genetically based cell-clusters (*t*-SNE plot) obtained from a total of 20 neocortical and hippocampal brain areas. Data from the Allen Institute as published previously (Hodge et al., 2019) and obtained and analysed in *CytosploreViewer* (https://viewer.cytosplore.org/). Relevant cell-clusters that show expression are labelled. <u>Abbreviations</u>: GC, granule cell (glutamatergic); IN, interneuron (GABAergic neuron); PC, pyramidal cell (glutamatergic neuron); L4/5/6, neocortical layer 4/5/6; HC, hippocampus, CA1/CA3, cornu ammonis region 1/2 of the hippocampus; DG, dentate gyrus region of the hippocampus; PV, parvalbumin positive; SST, somatostatin positive; VIP, vasoactive intestinal peptide positive; Lamp5, interneurons positive for Lamp5, Adarb5 or Scng.

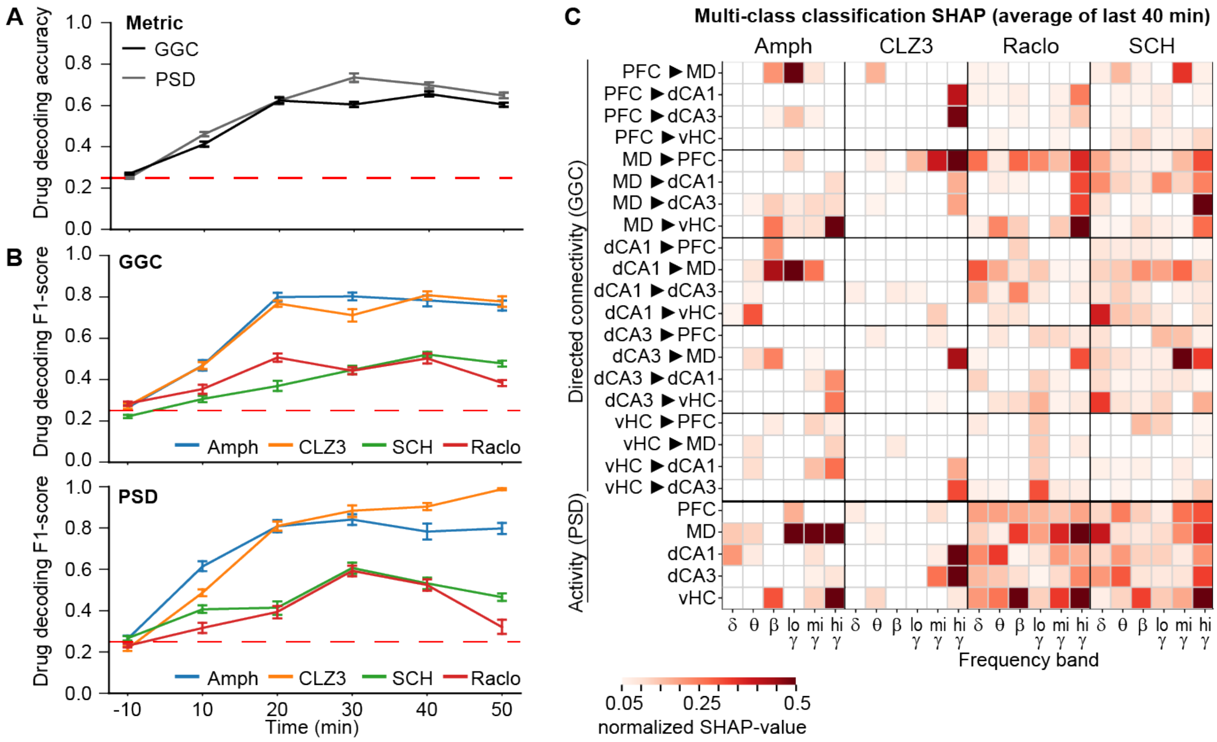

Supplementary Figure 6. Multi-class classification of all 4 compounds. (**A**) Drug decoding accuracy using either GGC-connectivity or PSD-activity and discriminating all four compounds at once in a multi-class classification shown over 10-min intervals. The lower dose of clozapine has not been used for this analysis to avoid confounding the factor of drug identity with that of dose. (**B**) F1-score indicating how well each compound can be discriminated against the other three using either GGC (top) or PSD (bottom). Red line indicates chance level (0.25). No statistics applied. (**C**) Average normalized SHAP-values from the multi-class classifier. Note that the frequency-subspace method, and hence accuracy-based weighing of SHAP values, have *not* been applied for this analysis.

Supplementary references

Akiba, T., Sano, S., Yanase, T., Ohta, T., and Koyama, M. (2019). Optuna: A Next-generation Hyperparameter Optimization Framework. doi: 10.48550/arXiv.1907.10902

Baccala, L. A., Sameshima, K., and Takahashi, D. Y. (2007). Generalized Partial Directed Coherence., in *2007 15th International Conference on Digital Signal Processing*, 163–166. doi: 10.1109/ICDSP.2007.4288544

Bokil, H., Andrews, P., Kulkarni, J. E., Mehta, S., and Mitra, P. P. (2010). Chronux: A platform for analyzing neural signals. *J. Neurosci. Methods* 192, 146–151. doi: 10.1016/j.jneumeth.2010.06.020

Delorme, A., and Makeig, S. (2004). EEGLAB: an open source toolbox for analysis of single-trial EEG dynamics including independent component analysis. *J. Neurosci. Methods* 134, 9–21. doi: 10.1016/j.jneumeth.2003.10.009

Delorme, A., Mullen, T., Kothe, C., Akalin Acar, Z., Bigdely-Shamlo, N., Vankov, A., et al. (2011). EEGLAB, SIFT, NFT, BCILAB, and ERICA: New Tools for Advanced EEG Processing. *Comput. Intell. Neurosci.* 2011, e130714. doi: 10.1155/2011/130714

Geweke, J. (1982). Measurement of Linear Dependence and Feedback Between Multiple Time Series. *J. Am. Stat. Assoc.* 77, 304–313. doi: 10.2307/2287238

Hodge, R. D., Bakken, T. E., Miller, J. A., Smith, K. A., Barkan, E. R., Graybuck, L. T., et al. (2019). Conserved cell types with divergent features in human versus mouse cortex. *Nature* 573, 61–68. doi: 10.1038/s41586-019-1506-7

Kapanaiah, S. K. T., and Kätzel, D. (2023). Open-MAC: A low-cost open-source motorized commutator for electro- and opto-physiological recordings in freely moving rodents. *HardwareX* 14, e00429. doi: 10.1016/j.ohx.2023.e00429

Korzeniewska, A., Crainiceanu, C. M., Kuś, R., Franaszczuk, P. J., and Crone, N. E. (2008). Dynamics of event-related causality in brain electrical activity. *Hum. Brain Mapp.* 29, 1170–1192. doi: 10.1002/hbm.20458

Lundberg, S. M., Erion, G., Chen, H., DeGrave, A., Prutkin, J. M., Nair, B., et al. (2020). From local explanations to global understanding with explainable AI for trees. *Nat. Mach. Intell.* 2, 56–67. doi: 10.1038/s42256-019-0138-9

Molnar, C. (n.d.). *Interpretable Machine Learning*. Available at: https://christophm.github.io/interpretable-ml-book/ (Accessed October 24, 2021).

Mullen, T. (2010). Source Information Flow Toolbox (SIFT) - An Electrophysiological Information Flow Toolbox for EEGLAB. Available at: https://sccn.ucsd.edu/githubwiki/files/eeglab2011_tm_sift.pdf

shap.TreeExplainer — SHAP latest documentation (n.d.). Available at: https://shap-lrjball.readthedocs.io/en/latest/generated/shap.TreeExplainer.html# (Accessed February 7, 2024).

Siegle, J. H., López, A. C., Patel, Y. A., Abramov, K., Ohayon, S., and Voigts, J. (2017). Open Ephys: an open-source, plugin-based platform for multichannel electrophysiology. *J. Neural Eng.* 14, 045003. doi: 10.1088/1741-2552/aa5eea

Strahnen, D., Kapanaiah, S. K. T., Bygrave, A. M., and Kätzel, D. (2021). Lack of redundancy between electrophysiological measures of long-range neuronal communication. *BMC Biol.* 19, 24. doi: 10.1186/s12915-021-00950-4
